# Platinum group metals for nuclear medicine, a luxurious dream or the future of imaging and therapy: a review

**DOI:** 10.3389/fnume.2025.1656374

**Published:** 2025-09-11

**Authors:** Daniel G. Racz, Ivis F. Chaple

**Affiliations:** Department of Nuclear Engineering, University of Tennessee, Knoxville, TN, United States

**Keywords:** platinum group metals, platinum, palladium, rhodium, osmium, iridium, ruthenium, nuclear medicine

## Abstract

Platinum group metals (PGMs) consist of six transition metals: platinum (Pt), palladium (Pd), rhodium (Rh), osmium (Os), iridium (Ir), and ruthenium (Ru). PGMs have been used notably in industrial, electronic, and medical applications. For example, Ir-192 is often utilized in industry to detect structural defects in metal and assess pipeline integrity. Pd-104 is irradiated to produce Pd-103 seeds, used for prostate cancer treatment. Other isotopes of elements in this group can be sourced to facilitate critical applications, discussed in this review. Due to their unique chemical and nuclear properties, these metals may be promising candidates for various nuclear medicine applications, including diagnostic imaging via Positron Emission Tomography (PET), Single Photon Emission Computed Tomography (SPECT) and Targeted Radionuclide Therapy (TRT). This review will explore PGMs in nuclear medicine, focusing on their production routes, nuclear characteristics, and suitability for past and future development of radiopharmaceuticals. We will highlight methods for radiochemical separation and purification of each radionuclide, discussing potential challenges and emphasizing the need for further research to ensure sustainability. As the demand for advanced nuclear medicine techniques continues to grow, PGMs may play a significant role in addressing current challenges in the field. We will discuss several radionuclides of interest to nuclear medicine including ^191^Pt, ^193m^Pt, ^195m^Pt, ^103^Pd, ^109^Pd, ^103m^Rh, ^105^Rh, ^191^Os, ^192^Ir, ^97^Ru, and ^103^Ru.

## 1 Introduction

Cancer remains one of the leading global health concerns, with an estimated 19.3 million new cancer cases and 10 million cancer-related deaths worldwide in 2020 (1), and projections are expected to rise by 60% within the next two decades (2). The increase incidence of cancer necessitates the development of innovative approaches towards both cancer diagnosis and treatment. Nuclear medicine provides powerful tools for both diagnostic imaging and therapy. Techniques such as position emission tomography (PET) and single photon emission computed tomography (SPECT) offer functional imaging capabilities that are crucial for accurately detecting cancerous cells. By administering a radiopharmaceutical that targets cancer-specific cells, nuclear medicine imaging can detect the radiation emitted from the radiopharmaceuticals, enabling personalized treatment planning. Therefore, these imaging modalities are crucial in clinical practice, guiding oncologists in selecting optimal conditions to improve patient outcomes. Beyond diagnostics, targeted radionuclide therapy (TRT) has been utilized as a cornerstone in oncology care. Radionuclides have also played vital roles alongside external beam radiation therapy (EBRT). For example, ^192^Ir sources are widely used in high dose rate brachytherapy for many types of cancers (3), and ^106^Ru is used as a standard eye-preserving treatment for uveal melanoma (4). Whether a radionuclide is used in either diagnostic imaging or radiotherapeutics, the continued exploration of their application is of key importance towards nuclear medicine advancement.

The discovery of the six platinum group metals (PGMs)—platinum (Pt), palladium (Pd), rhodium (Rh), osmium (Os), iridium (Ir), and ruthenium (Ru)—spanning from the sixteenth to the nineteenth centuries, laid the foundation for advancements across diverse fields. Platinum was first discovered in the sixteenth century in Colombia's Choco district, where four additional PGMs—palladium, rhodium, osmium, and iridium—were classified three centuries later by William H. Wollaston and Smithson Tenant (5). Wollaston discovered palladium and rhodium by refining platinum ores, where Tennant discovered iridium and osmium in the residues (5). The sixth PGM, ruthenium, was extracted by Karl Klaus in 1844 from platinum residues, and named after “Ruthenia” (Latin for Russia) (5, 6). Today, these elements are integral not only in everyday technologies such as electronics and automotive catalysts but also in innovative medical treatments, where their unique properties—chemical, physical, nuclear—enable breakthroughs in cancer therapies, imaging, and radiopharmaceuticals.

Platinum-based compounds, especially in the oxidation states of 2+ and 4+, have been widely, and successfully, utilized for the treatment of many types of cancers (2, 7). The two most notable platinum-based chemotherapy agents are cisplatin and carboplatin, although there are several other variations of these drugs which are mainly characterized by a central Pt atom surrounded by atoms of Cl, NH_2_, CH_3_, and others. The toxicity of these compounds has become a major limitation in their use, as it can to some extent, have a negative effect on all organs (8). New strategies, such as the development of Pt(IV) prodrugs have been described to overcome the effects that diminish clinical outcomes (7). Palladium has shown similarities between the coordination chemistry of Pd(II) and Pt(II) compounds, advocating studies to implement Pd(II) complexes as antitumor drugs (9–11). Furthermore, described in Lazarevic et al. (11), Pd(II) compounds exchange ligands 10^4^–10^5^ times faster than corresponding Pt(II) analogs—with various compounds showing anti-inflammatory, antimicrobial (12), antitumor (13), antibacterial, antiviral, and antifungal capabilities (14). Ma et al. (15) stated that research into bioactive rhodium complexes are warranted and may eventually lead to the discovery of drugs with distinct mechanisms of action compared to traditional platinum or ruthenium-based therapeutics. Due to the well-known toxicity of OsO_4_, osmium's utility in medicinal chemistry has been less explored; however, the implementation of novel ligands and the diverse coordination geometries and oxidation states of this metal has led to further development (16)—with several papers discussing potential osmium anticancer agents (17, 18). Konkankit et al. (16) highlighted a surge in the application of iridium complexes as anticancer agents and imaging probes, for example, octahedral cyclometalated iridium complexes, along with complexes targeting DNA and inhibiting specific proteins. Ruthenium has emerged as a “next-generation” therapeutic metal, while offering several advantages over Pt-based drugs, including electronic structure, tunable redox properties, and a relatively low toxicity profile contributing to its increasing importance (2, 7).

In addition to PGM compounds being utilized in traditional medicine, PGMs also contribute towards nuclear medicine due to useful nuclear decay properties essential for imaging or radionuclide therapy. In this review, a detailed evaluation of select PGM radionuclides, tracing their past developments and current successes or challenges towards advancement in nuclear medicine. Their nuclear properties will also be discussed, along with reviewing production routes and radiochemical separation methods that enable high purity radionuclide preparation. Moreover, key results from either pre-clinical or clinical studies, are reported, along with a discussion on how PGMs may be added as novel tools to the toolkit of modern nuclear medicine. Through this review, we aim to illustrate whether PGMs are a luxurious dream or a key to the future of molecular imaging and radionuclide therapy.

## 2 Platinum and platinum-based radionuclides

Naturally occurring platinum isotopes include: ^190^Pt (0.012%,), ^192^Pt (0.782%), ^194^Pt (32.864%), ^195^Pt (33.77%), ^196^Pt (25.21%), and ^198^Pt (7.356%) (19). For this review, we will focus on ^191^Pt, ^193m^Pt, and ^195m^Pt, as other radioisotopes (^188^Pt, ^189^Pt, ^197^Pt) have not been widely studied.

### 2.1 Platinum-191, ^191^Pt

Due to its nuclear decay properties, ^191^Pt may be suitable for Auger electron therapy. This radionuclide has a half-life of 2.83 d and decays 100% by electron capture (EC), with notable *γ*-ray energies of 538.9 keV (*I_γ_* = 13.7%) and 465.5 keV (*I_γ_* = 3.5%) (20).

#### 2.1.1 Production and radiochemical separation of ^191^Pt

Multiple production routes for ^191^Pt have been explored using either osmium or iridium targets bombarded with protons, deuterons, or α-particles highlighted in Table 1. Bonardi et al. (21) produced no-carrier-added (n.c.a.) ^191^Pt—which complemented earlier work from Parent et al. (22) and Sharma and Smith (23)—while achieving 170 MBq/μg with decontamination factors of >10^6^ via two optimized radiochemical separations (Sn(II)/ether vs. NH_2_OH/dithizone extraction). Obata et al. (20) measured excitation functions, finding peak cross sections of ∼623–635 mb for ^191^Pt at ∼26–32 MeV, with theoretical thick-target yields of ∼108–192 MBq/μA-h for both proton and deuteron irradiation using ^nat^Ir or ^193^Ir targets. Furthermore, they noted ∼25 MeV protons as the optimal energy, though advanced target dissolution methods were needed due to iridium exhibiting superior resistance to acid (20). Obata et al. (24) addressed this by using an alkali-fused Ir target and *in situ* HCl digestion, followed by solvent extraction and anion exchange, yielding 17.4 ± 1.1 MBq/μA-h at EOB (7.1 ± 0.4 MBq/μA-h post separation) with >99% radionuclidic purity.

**Table 1:** Production pathways for platinum-based radionuclides.

Radionuclide	Nuclear reaction	Flux/Energy	References
^ 191^Pt	^nat^Os(α,xn)^191^Pt	E_max_ = 38 MeV	(21, 260)
	^192^Os(^3^He,4n)^191^Pt	36 → 25 MeV	(261)
	^nat^Ir(p,xn)^191^Pt	E_max_ = 30 MeV	(20–22, 24, 262)
	^nat^Ir(d,xn)^191^Pt	E_max_ = 40.3 MeV	(20, 260, 263)
^ 193m^Pt	^192^Os(α,3n)^193m^Pt	E_max_ = 39 MeV	(44, 45, 51)
	^192^Pt(n,*γ*)^193m^Pt	4 × 10^14^ n cm^−2^ s^−1^	(52)
^ 195m^Pt	^193^Ir(n,γ)^194^Ir(n,γ)^195m^Ir → ^195m^Pt	1–2.5 × 10^15^ n cm^−2^ s^−1^	(57, 59)
	^194^Pt(n,γ)^195m^Pt	3–8.5 × 10^13^ n cm^−2^ s^−1^	(26, 59, 63, 64, 66)
	^195^Pt(n,n’)^195m^Pt		(60)
	^192^Os(α,n)^195m^Pt	28 → 16 MeV	(45)
	^197^Au(γ,n)^195m^Pt	E_max_ = 34 MeV	(67)

#### 2.1.2 Applications of ^191^Pt

**Figure 1:**
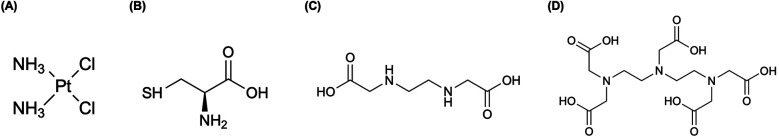
A select subset of a ^191^Pt complex and chelators discussed in this section are highlighted. **(A)** The chemotherapy agent, *cis-*diamminedichloroplatinum(II) (cisplatin) is widely used in the treatment of various forms of carcinomas and sarcomas, which was radiochemically synthesized with ^191^Pt ([^191^Pt]-cisplatin) for investigation (25–28, 30). The complex is represented as elemental platinum. Obata et al. (31) compared ^191^Pt coordination to **(B)** amino acid cysteine (Cys), and both multidentate chelators, **(C)** ethylenediaminediacetic acid (EDDA) and **(D)** diethylenetriaminepentaacetic acid (DTPA).

Areberg et al. (25) demonstrated the first use of [^191^Pt]cisplatin (Figure 1A) for tumor imaging. Fourteen patients received [^191^Pt]cisplatin (≥99% radionuclidic purity)—synthesis based on the work reported by Hoeschele et al. (26)—and showed clear gamma-camera visualization of tumors in multiple anatomical sites (25). Building on this, the same group (27) reported organ-specific absorbed and effective doses for [^191^Pt]cisplatin (and ^193m^Pt/^195m^Pt analogs)—advancing beyond earlier whole-body mean dose calculations by Lange et al. (28).

Recent work has leveraged the auger electrons emitted from ^191^Pt towards targeted therapy. Obata et al. (29) developed a resin-based method to isolate n.c.a. [^188,189,191^Pt]Pt(II)Cl_4_^2−^, and a one-pot radiosynthesis of [*Pt]cisplatin, yielding 30%–40% without intermediate evaporation. Using tracer-level [^189,191^Pt]cisplatin, Obata et al. (30) showed only 0.6% overall cell uptake in cells, yet ∼20% of internalized platinum localized to the nucleus and ∼2% bound covalently to DNA (0.28 ± 0.02% ID/mg) (30). Single-cell assays confirmed that auger electrons caused direct DNA double-strand breaks, validating [^189,191^Pt]cisplatin as an extremely localized therapeutic with minimal systemic toxicity (30). Obata et al. (31) compared ^191^Pt coordination to Cys, DTPA, EDDA (Figures 1B–D) to evaluate the *in vitro* behavior to analogous ^111^In-labeled (t_1/2_ = 2.8 d, 100% EC) agents (31–34). They demonstrate that free ^191^PtCl_4_^2−^ undergoes rapid thiol coordination with Cys, significantly reducing protein binding at 60°C (∼10%) compared to 45°C (∼42%) (31). In contrast, labeling with DTPA and EDDA resulted in moderate radiochemical yields (70%–80%) and reduced protein binding only to ∼42% and ∼30%, respectively (31). Furthermore, ^191^Pt was complexed with the DNA-targeting dye Hoechst33258 via DTPA ([^191^Pt]Pt-DTPA-Hoechst33258; >95% radiochemical purity) and Cys ([^191^Pt]Pt-Cys-Hoechst33258; ∼90% radiochemical purity) to compare with [^111^In]In-DTPA-Hoechst33258 (>95% radiochemical purity) and found both ^191^Pt-based complexes displayed one order of magnitude greater DNA-binding than the ^111^In analog (31). Notably, [^191^Pt]Pt-Cys-Hoechst33258 induced DNA damage more effectively than its DTPA counterpart, suggesting that thiol-based ^191^Pt labeling enhances delivery to DNA and elevates therapeutic potential (31).

Obata et al. (35) conjugated ^191^Pt to a oncogene MYCN-specific pyrrole-imidazole polyamide (PIP) scaffold (^191^Pt-MYCN-PIP) bearing Cys, tri-arginine (R3) for cellular penetration (36), and a fluorescent compound coumarin (GCC-Cys-R3-coumarin control, ^191^Pt-GCC-PIP). The MYCN gene is a transcription factor that is amplified in human neuroblastoma and is related to the patient's prognosis (35). Noted in Obata et al. (35), targeting cancer-related genes with PIPs have been utilized in preclinical studies with mice and marmosets (37, 38), along with developments of MYCN-targeting PIP in Yoda et al. (39) showed promising specific targeting ability and therapeutic effects. With 50%–70% radiochemical yield and >95% radiochemical purity, ^191^Pt-MYCN-PIP achieved ∼10-fold higher uptake and DNA-binding in MYCN-amplified vs. non-amplified neuroblastoma cells, and reduced MYCN expression *in vitro* (35). Omokawa et al. (40) synthesized a sugar-conjugated platinum complex, FGC-Pt (*cis*-dichloro[(2-fluoro-α-_D_-glucopyranosidyl)propane-1,3-diamino-2-propyl]platinum) (41) and labeled it with n.c.a. ^191^Pt by either direct activation (61.7% radiochemical purity) or post-labeling of neutron-activated [^191^Pt]K_2_PtCl_4_ (14.5 ± 7.3% radiochemical yield; 93.8% radiochemical purity), with the latter method providing significantly higher yield and purity. In healthy mice, both [^191^Pt]FGC-Pt preparations showed almost identical biodistribution at 24 h—and γ-counting correlated with ICP-MS measurements (*r* = 0.92, *p* < 0.05), confirming their utility for quantitative imaging (40). Most recently, Obata et al. (42) developed a PSMA-targeting ^191^Pt-trithiol complex showing a 46-fold uptake advantage in PSMA^+^ vs. PSMA^−^ tumors (*in vitro*), outperforming the Cys-based analog (2.2 ± 0.3).

### 2.2 Platinum-193 m, ^193m^Pt

Platinum-193 m is a metastable isomer of platinum-193 that may be useful for Auger electron therapy. This radionuclide is attractive due to emitting around 26 Auger electrons per decay and has a half-life of 4.33 days (43).

#### 2.2.1 Production and radiochemical separation of ^193m^Pt

The production routes to obtain ^193m^Pt are shown in Table 1. Uddin et al. (44) measured the experimental excitation function for the ^192^Os(α,3n)^193m^Pt reaction—building on previous work by Hilgers et al. (45)—reporting a peak cross section of 1.47 ± 0.19 b (66.63 keV x-ray) and 1.53 ± 0.21 b (135.5 keV γ-ray), both at 36.4 ± 0.2 MeV. As the authors noted, several methods for the dissolution of osmium had been reported (45–47). An optimized electrolytic technique was carried out to prepare highly enriched ^192^Os, where the authors noted, low electrodeposition yields were minimal to this point (44). Jones et al. (48) reported a maximum deposition of 9.5% at pH 13—which encouraged the authors to focus on this effort. Chakrabarty et al. (47) on the other hand, reported a high yield of ∼80% for an isotopically enriched osmium sample, where efforts by Uddin et al. (44) were devoted to optimizing the electrolytic deposition process. By using their electrolyte, a maximum electrodeposition yield of ∼75% at pH 12.8 was achieved for the enriched osmium, with 15% lower for natural osmium. Adopting radiochemical separation techniques from Bonardi et al. (21) and Hilgers et al. (45), Uddin et al. (44) oxidized the osmium sample with the Ni backing in concentrated nitric acid and evaporated out the liquid. The OsO_4_ was distilled and trapped in 4.7 N KOH, while the residual Pt was dissolved in 3 N HCl, and reduced from Pt(IV) to Pt(II) with SnCl_2_. The [Pt(SnCl_3_)_5_]^3−^ anion was extracted into the ether phase, achieving a radiochemical yield of 80%–96% across 20 individual osmium samples (44). Compared to Hilgers et al. (45) and predictions from nuclear model codes [TALYS (49) and STAPRE (50)], the measured excitation functions from (44) showed excellent agreement across the energy range. Integral yield calculations 1 μA for 1 h yielded ∼10 MBq/μA-h of ^193m^Pt and ∼0.06 MBq/μA-h of ^195m^Pt within the optimal energy window of 40→30 MeV, establishing ^192^Os(α,3n)^193m^Pt as the most effective cyclotron-based route for producing clinically relevant quantities of ^193m^Pt (44).

Uddin et al*.* (51) demonstrated a small-scale, cyclotron-based production of ^193m^Pt via ^192^Os(α,3n)^193m^Pt reaction, achieving 99% radionuclidic purity and a specific activity of 1 GBq/μg ^193m^Pt, effectively overcoming the limitations of low specific activity associated with reactor-based (n,γ) production on ^192^Pt targets as highlighted by Azure et al. (52). Target dissolution and OsO_4_ distillation, previously reported in Hilgers et al. (45) and Uddin et al. (44), combined with a SnCl_2_-ether extraction sequence developed by Ahmed and Koch (53) and Koch and Yates (54), enabled 85% recovery of enriched Os and 90% radiochemical yield of ^193m^Pt (51). The experimental batch yield at EOB was ∼10 MBq using a 1.6 μA beam for 3 h, corresponding to ∼40% of the theoretical value predicted from the excitation function of the ^192^Os(α,3n)^193m^Pt reaction (44, 51). In contrast, (n,γ) production using 5 mg of 57% enriched ^192^Pt (ϕ = 4 × 10^14^ n cm^−2^ s^−1^; 7 d) yielded 3 GBq with a specific activity of only 0.6 MBq/μg ^193m^Pt (51, 52). Moreover, α-induced production results in minimal ^195m^Pt impurity (0.5%) compared to the (n,γ) route (∼12%), emphasizing its suitability for scalable, high-purity Auger-electron radionuclide production (51, 52).

#### 2.2.2 Applications of ^193m^Pt

**Figure 2:**
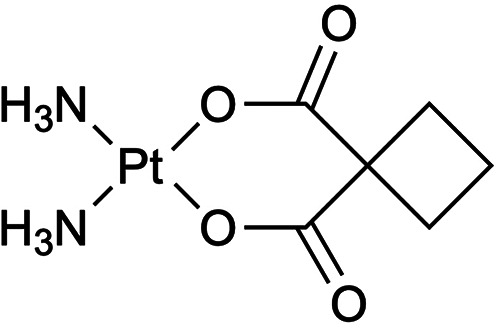
An analog of cisplatin, *cis*-diammine(1,1-cyclobutanedicarboxylato)-platinum(II) (carboplatin, paraplatin®) has demonstrated to strongly diminish renal toxicity and other associated deleterious physiological phenomena, along with clearance of the drug from the body is much faster than for cisplatin (52). We present the complex structure using elemental platinum; however, Azure et al. (52) radiochemically synthesized carboplatin with ^193m^Pt ([^193m^Pt]carboplatin) (52).

**Figure 3:**
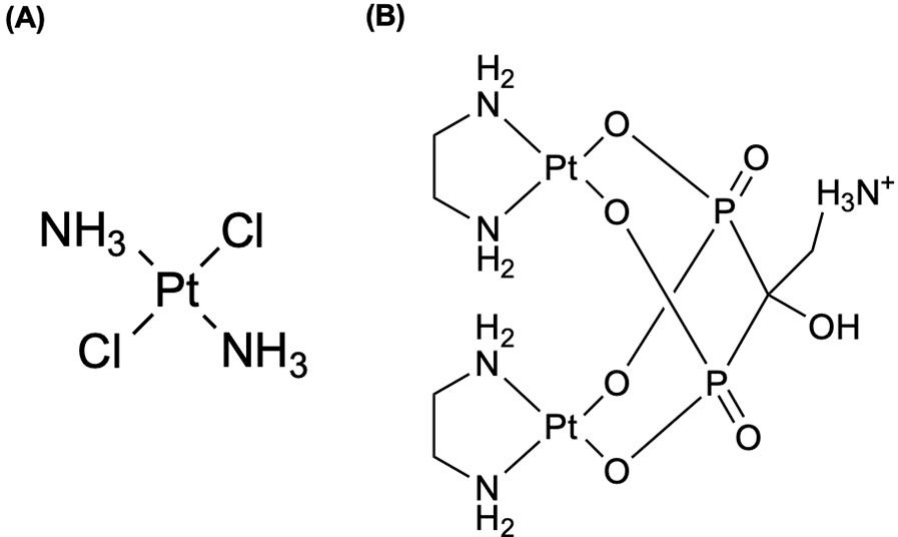
**(A)** The complex of *trans*-dichlorodiammineplatinum(II) (transplatin) was radiochemically synthesized with radioactive platinum ([^195m^Pt]-transplatin) in Howell et al. (56) to be utilized as an antitumor drug (56). **(B)** Radioactive bisphosphonate-functionalized platinum (^195m^Pt-BP) complexes were investigated to specifically accumulate in intratibial bone metastatic lesions in mice (74–76). Both complexes are presented as elemental platinum.

Lange et al. (55) performed the radiosynthesis of cisplatin labeled with ^193m^Pt and subsequent biodistribution on rabbits and mice. From their findings following intravenous injection, most of the activity accumulated in the kidneys, urine, and liver, with rapid excretion of the radiolabeled complex (79% eliminated by 24 h) (55). A year later, the same group (28), performed distribution studies and dose calculations for ^193m^Pt and ^195m^Pt and reported similar biodistribution results from the prior study, along with similar behavior with the ^195m^Pt-labeled analog (28). Azure et al. (52) performed the first microscale synthesis of carboplatin labeled with ^193m^Pt, reporting [^193m^Pt]carboplatin (Figure 2) uptake had saturated by 2–3 in V79 cells, and similar findings to [^195m^Pt]transplatin in Howell shown in Figure 3A (56). Notably, ∼70% of internalized ^193m^Pt was in the nucleus, with ∼60% of that bound to DNA (52)—substantially higher targeting than observed with ^195m^Pt (25% cellular radioactivity in nucleus, 42% bound to the DNA) (56).

### 2.3 Platinum-195 m, ^195m^Pt

Owing to its favorable nuclear decay properties, ^195m^Pt has been studied for its use in both nuclear medicine diagnosis and therapy. The radionuclide has a half-life of 4.02 d, emits low-energy photons (*E_γ_* = 98.85 keV, *I_γ_* = 11.4%), each disintegration releases 36 Auger electrons depositing around 25 keV within nm-μm distances in tissue (57, 58).

#### 2.3.1 Production and radiochemical separation of ^195m^Pt

High specific activity ^195m^Pt is best obtained via indirect reactor routes or enriched target irradiation, and all its production routes are shown in Table 1. Knapp et al. (59) produced n.c.a. ^195m^Pt by irradiating enriched ^193^Ir to produce ^195m^Ir (t_1/2_ = 3.67 h)—via ^193^Ir(n,γ)^194^Ir(n,γ)^195m^Ir—which then decays (*β*^−^) to ^195m^Pt while taking advantage of the high thermal flux of the High-Flux Isotope Reactor (HFIR) at Oak Ridge National Laboratory (ORNL) to surpass the specific activities achievable by direction ^194^Pt(n,γ) or ^195^Pt(n,n') routes (26, 59, 60). ^195m^Pt was separated from bulk Ir via thiourea-HCl elution on cation resin—where methods were previously reported by Siegfried et al. (61) and Berg and Senn Jr (62).—yielding high purity of ^195m^Pt (59). Hilgers et al. (45) measured the ^192^Os(α,n)^195m^Pt reaction, reporting a maximum cross section of 4.4 ± 0.7 mb at 22.1 ± 0.7 MeV, and projected ∼0.09 GBq yield—about an order of magnitude lower than reactor methods (63). Vosoughi et al. (64) irradiated ^nat^Pt in a reactor (3 × 10^13^ n cm^−2^ s^−1^, 30 h, 5 MW power), obtaining 16.28 MBq of ^195m^Pt. The product was allowed to decay for 48-h due to short-lived Au/Pt impurities and solvent extraction separation was performed following an established method by Vimalnath et al. (65), they obtained radiochemical yield and separation efficiency of ≥99% and 99.4%, respectively (64). However, specific activity was only ∼0.8 MBq/mg, much lower compared to the reported <37 MBq/mg (59) and 15.9 MBq/mg (66) that were achieved with enriched ^194^Pt targets at ORNL and SAFARI-1 reactors, respectively (64). Bodnar et al. (67) aimed to develop a method of preparation of ^195m^Pt with high specific activity via a photonuclear reaction. Obtaining ^195m^Pt via the ^197^Au(*γ*,np)^195m^Pt reaction, they implemented a novel technique for gold extraction and produced high specific activity ^195m^Pt >1 Ci/mg (67). Wawrowicz and Bilewicz (57) tested the double-neutron capture approach but proved it to be impractical due to an unknown second-step cross section and difficult target dissolution, yielding <10% recovery. Therefore, until nuclear data and chemical processing improves, double-capture routes offer no advantage (57).

#### 2.3.2 Applications of ^195m^Pt

Leveraging reactor-produced n.c.a. ^195m^Pt, Zeevart et al. (66) prepared [^195m^Pt]cisplatin for a Phase 0 clinical trial on healthy patients (66). Using an optimized synthesis—building on work by Smith (68)—[^195m^Pt]cisplatin was obtained in >95% radiochemical yield (^195m^Pt and ^197^Pt combined), with co-produced impurities (^192^Ir, ^191^Pt, Au isotopes) below detection (66). Sathekge et al. (69) obtained whole-body planar scans and SPECT/CT images up to 144 h post-[^195m^Pt]cisplatin injection in five volunteers. Bodnar et al. (67) also optimized the radiosynthesis of ^195m^Pt-cisplatin from earlier works of Chernyaev (70) and Dykiy et al. (71) for *in vitro* and *in vivo* evaluation. They confirmed induced necrosis and apoptosis *in vitro* at mass doses over five orders of magnitude lower than conventional cisplatin doses (67, 70, 71). In mice with Ehrlich tumors, a single [^195m^Pt]cisplatin dose achieved 65% tumor growth inhibition—and 100% animal survival—vs. 35% inhibition by conventional cisplatin (67).

Apart from cisplatin analogs, Aalbersberg et al. (72) conducted a preclinical evaluation of ^195m^Pt SPECT using NanoSPECT/CT and U-SPECT^+^/CT scanners following thermal neutron irradiation of ^194^Pt in the High Flux Reactor (HFR) in Petten, the Netherlands. They achieved sub-millimeter resolution and linear quantification over a wide activity range (0.035–4.36 MBq), confirming accurate *in vivo* Pt distribution measurements (72). SPECT-based quantification, calibrated using a ^195m^Pt dilution series, correlated strongly with ex vivo gamma-counting and graphite-furnace atomic absorption spectroscopy (GF-AAS), validating accurate *in vivo* quantification of platinum biodistribution (72). Although the study validated the feasibility of ^195m^Pt SPECT in small animals, the authors noted limitations including low specific activity 3–4 MBq per injection, small sample size, and the need to improve purification methods to extend imaging with radiolabeled cisplatin (72). Muns et al. (73) characterized a metal-organic linker, [ethylenediamineplatinum(II)]^2+^ (called *Lx*) with antibody-drug conjugates (ADCs) for *in vivo* stability and tumor targeting using ^195m^Pt and ^89^Zr (t_1/2_ = 78.36 h). Nearly identical ^195m^Pt and ^89^Zr biodistributions in tumor-bearing mice confirmed the *in vivo* stability of the Pt(II)-histidine coordinative bond within *Lx* (73). However, the amounts of platinum incorporated into *Lx*-based ADCs and the specific activity of ^195m^Pt were too low to support preclinical or clinical SPECT imaging studies (73).

Nadar et al. (74) synthesized a n.c.a ^195m^Pt-BP complex, shown in Figure 3B, to achieve bone-targeting Auger-electron therapy. This complex was introduced previously by Margiotta et al. (75). In healthy C57BL/6N mice (2.5 mM Pt, 24 h), ICP-MS showed a 4.5-fold higher uptake in hard tissue (12.18 ± 0.56%ID/g) vs. its bisphosphonate-free precursor Pt(NO_3_)_2_(en) (2.69 ± 0.26%ID/g), and accomplished reducing off-target retention in many organs including the kidney (5.70 ± 0.15 vs. 3.38 ± 0.28%ID/g) (74). Pt-BP also induced minimal Pt-DNA adduct formation (<0.5% of total Pt in most tissues; kidney: 2.8%, spleen: 1.4%) compared to the precursor (kidney: 4.8%, spleen: 9.8%), confirming that bisphosphonate conjugation both enhances bone selectively and spares healthy tissues for DNA damage (74). In micro-SPECT/CT studies, ^195m^Pt-BP rapidly localized to growth plates, whereas ^195m^Pt(NO_3_)_2_(en) accumulated specifically in soft tissues (74). Laser ablation ICP-MS (LA-ICP-MS) further validated 73.5% co-localization of ^195m^Pt-BP, showing almost a four-fold increase accumulation of Pt in bone compared to the precursor—highlighting its specific bone-binding mechanism (74). In a subsequent study, Nadar et al. (76) treated mice with intratibial bone tumors using ^195m^Pt-BP and [^195m^Pt]cisplatin. ^195m^Pt-BP exhibited significantly higher and sustained accumulation in metastatic lesions with 2.8–3.3-fold higher uptake than the contralateral tibia, indicating selective targeting (76). In contrast, ^195m^Pt-cisplatin exhibited lower uptake (≤3.7%ID/g) with no evidence of lesion selectivity at any time point (76). Therapeutic efficacy was assessed via γ-H2AX staining—a biomarker specific for double-strand DNA breaks—revealing that ^195m^Pt-BP induced a 4.6-fold greater fraction of γ-H2AX-positive tumor cells (1.66 ± 0.4%) compared to ^195m^Pt-cisplatin (0.36 ± 0.1%) and an 11-fold increase over non-radioactive Pt-BP (0.15 ± 0.1%) (76). These results confirm that bone-targeted ^195m^Pt-BP delivers Auger radiation directly to tumor-associated bone lesions with superior efficacy compared to [^195m^Pt]cisplatin (76).

Most recently, de Roest et al. (77) explained [^195m^Pt]cisplatin uptake in cisplatin-sensitive and -resistant head-and-neck cancer models. They found that cisplatin-resistant HNSCC cell line (VU-SCC-OE) accumulated more [^195m^Pt]cisplatin in DNA and exhibited greater capacity to repair cisplatin-induced crosslinks compared to the cisplatin-sensitive HNSCC cell line (VU-SCC-1131), with a DNA retention ratio of 3.4 vs. 1.45 (77). The authors concluded that [^195m^Pt]cisplatin imaging is not predictive of tumor sensitivity to cisplatin but may serve as a tool for assessing cisplatin-related off-target toxicity (77).

## 3 Palladium and palladium-based radionuclides

There are six naturally occurring stable isotopes of palladium: ^102^Pd (1.0%), ^104^Pd (11.0%), ^105^Pd (22.2%), ^106^Pd (27.3%), ^108^Pd (26.7%), and ^110^Pd (11.8%) (19). Radioisotopes of palladium include ^100^Pd, ^103^Pd, ^107^Pd, and ^109^Pd, in this review we will discuss ^103^Pd and ^109^Pd.

### 3.1 Palladium-103, ^103^Pd

^103^Pd (t_1/2_ = 16.99 d) is a therapeutic radionuclide that has been used in brachytherapy for the treatment of prostate cancer, mostly used as a metal seed or stent (78, 79). The radionuclide decays to ^103m^Rh by electron capture, which then de-excites through internal transition (IT) to stable ^103^Rh. ^103^Pd emits x-rays and Auger electrons due to the EC and IT decays, which makes ^103^Pd suitable for internal radiotherapy (79).

#### 3.1.1 Production and radiochemical separation of ^103^Pd

A variety of production methods exist for ^103^Pd, including reactor- and accelerator-based routes which is described in Table 2. Sudar et al. (80) reported a maximum cross-section of 505 ± 26 mb at 10.05 ± 0.19 MeV (via x-ray measurements) and identified the optimal energy range for maximizing specific cross-sections (300–500 mb) and yields to be between 8 and 12 MeV. The authors compared between neutron-counting studies—including those by Albert (81), Johnson et al. (82), and Hansen and Albert et al. (83)—and activation measurements—Blaser et al. (84), Harper et al. (85), Treytl and Caretto (86), Mukhammededov and Vasidov (87), and Hermanne et al. (88)—from energies 2.8–400 MeV, confirming good agreement across studies, with discrepancies at lower energies mainly attributing to systematic uncertainties and differences in target preparation (80). Building on this, Hussain et al. (79) provided a comprehensive evaluation of all accelerator-based production routes for n.c.a. ^103^Pd, integrating six reaction channels (89–95) reported in Table 2 using EXFOR data and key literature sources, and by normalizing the raw measurements with three nuclear-reaction codes (STAPRE (50), TALYS (49), and EMPIRE (96)) to produce recommended excitation functions with 95% confidence limits. Furthermore, they investigated another indirect precursor of ^nat^Pd(p,x)^103^Ag→^103^Pd (97, 98) that can form up to 70% of total ^103^Pd via ^103^Ag decay but suffers from long-lived impurities and complex chemistry, limiting their large-scale clinical applicability (79).

**Table 2:** Production pathways for palladium-based radionuclides.

Radionuclide	Nuclear reaction	Flux/Energy	References
^ 103^Pd	^nat^Ag(p,x)^103^Pd	E_max_ = 100 MeV	(79, 89–91)
	^103^Rh(p,n)^103^Pd	E_max_ = 50 MeV	(79, 80, 101, 102)
	^103^Rh(d,2n)^103^Pd	E_max_ = 34 MeV	(79, 92, 93, 99)
	^100^Ru(α,n)^103^Pd	25 → 9 MeV	(79, 94, 95)
	^101^Ru(α,2n)^103^Pd	25 → 15 MeV	(79, 94, 95)
	^102^Ru(^3^He,2n)^103^Pd	34 → 7 MeV	(79, 94, 95)
	^nat^Pd(d,xn)^103^Ag → ^103^Pd	E_max_ = 20.5 MeV	(95, 98)
	^nat^Pd(p,x)^103^Ag → ^103^Pd	E_max_ = 37.3 MeV	(79, 97, 98)
^ 109^Pd	^108^Pd(n,γ)^109^Pd	3 × 10^13^ n cm^−2^ s^−1^	(78, 123)

Manenti et al. (99) optimized n.c.a. ^103^Pd production via the ^103^Rh(d,2n) reaction using a stacked-foil activations method at deuteron energies from 5 to 33 MeV on the JRC-Ispra and ARRONAX cyclotrons (beam currents 100–170 nA, 1 h irradiations). Experimental cross-sections rose steadily above the 3.62 MeV threshold, peaking at 1,261 ± 71 mb at 15.0 ± 0.4 MeV, and then declined gradually at higher energies (99). Comparison with prior data and models showed good agreement with Hermanne et al.'s (92) γ-ray measurements and close agreement with the recommended values of Hussain et al. (79), while Ditroi et al. (100) reported cross-sections up to 15% lower (99). Furthermore, EMPIRE-II and EMPIRE-3.2.2 (96) both reproduced the experimental curve within uncertainty, whereas TENDL-2015 (49) underestimated cross-sections above 10 MeV (99). Thick-target yields (TTYs) were computed from integrated thin-foil data, reporting that up to ∼12 MeV, deuteron-induced TTYs matched those of the ^103^Rh(p,n) route (99). Above 12 MeV, deuteron yields exceed proton yields by up to a factor of two—reflecting the higher (d,2n) cross-section at medium energies and marking deuteron beams as especially attractive for high-throughput production (99). Radionuclidic purity within the 5–33 MeV window is excellent as authors noted only ^101^Pd (t_1/2_ = 8.47 h) co-produces above its 22 MeV threshold, greatly simplifying post-irradiation separation (99). The higher stopping power of 13.3 MeV deuterons also reduces target mass, with a 188 μm Rh foil suffices for full absorption vs. 214 μm for 10.5 MeV protons, marginally easing radiochemical separation (99). Despite these advantages, high-energy deuteron cyclotrons remain scarce, which may constrain routine clinical-scale ^103^Pd production (99).

Ohya et al. (101) demonstrated an efficient method for producing no-carrier-added ^103^Pd, followed by radiochemical separation and target material recycling. The radiochemical separation incorporated a Bi-Rh alloying pretreatment at 500°C, enabling high-yield dissolution of the Rh target and achieving a 93 ± 4% dissolution efficiency (101). Following co-precipitation to remove Bi and palladium radionuclides—including ^100^Pd and ^103^Pd— a dimethylglyoxime (DMG)-based extraction, achieved 99 ± 1% yield (101). The radiopalladium was subsequently back-extracted from chloroform using aqueous ammonia, yielding 97 ± 2% of [^103^Pd(NH_3_)_4_]^2+^ (101). The entire process was completed within 3.5 h, yielding a ^103^Pd radiochemical yield of 87% and >99% radionuclidic purity (101). During the recycling process, 91 ± 3% of the Rh target was efficiently recovered with minimal Bi contamination (9 μg per 50 mg Rh) through cation exchange purification; therefore, providing a framework for clinical-scale ^103^Pd radionuclide production (101).

Krol et al. (102) presented the first feasibility study on the production of ^103^Pd via the ^103^Rh(p,n)^103^Pd reaction using cyclotron irradiation of a liquid target. By achieving an EOB activity of 1.03 ± 0.05 MBq (20.06 ± 0.97 MBq/μA) under optimized conditions (30 ± 0.5 μA, 1 h irradiation, 200 psi top up pressure, and 16.4 mg/ml metal-salt concentration), they demonstrated that liquid targets can reliably yield research-scale quantities of ^103^Pd suitable for radiochemistry (102). Furthermore, an anion-exchange separation using Dowex 1 × 8 resin with 1 M HNO_3_ for rhodium elution achieved a 90.1 ± 2.1% recovery from the irradiated target solution, while a 1:1 mixture of 0.5 M NH_3_ + NH_4_Cl for palladium elution resulted in a 103.8 ± 2.3% recovery—achieving a rhodium reduction factor of ∼10^6^ (102). More recently, Laouameria et al. (103) addressed previous limitations by developing a diffusion-driven extraction to separate ^103^Pd from its stable ^103^Rh target, relying on the metals' differing vapor pressures. Using their radionuclide separation equipment (RSE), they achieved an overall separation of 17 ± 2% and deposition yields of 77 ± 2% on Nb foil and 49 ± 2% on ZnO/W discs, respectively (103). Furthermore, using the ZnO/W disc substrate, the method produced 31.9 MBq EOB with a specific activity of 8.1 GBq/g, representing a streamlined alternative to traditional wet-chemistry approaches for Auger-emitter production (103).

#### 3.1.2 Applications of ^103^Pd

Blasko et al. (104) conducted a study on a cohort of 230 men with clinically T1-T2 prostate cancer treated exclusively with ^103^Pd brachytherapy. The study found an overall 9-year biochemical control rate of 83.5%, with PSA-only progression observed in just 4.3% of patients (104). The findings validated ^103^Pd brachytherapy as an effective and durable treatment option cross a range of risk groups, achieving high biochemical and clinical outcomes in patients with organ-confined prostate cancer (104).

Li et al. (105) developed an electroless plating method to fabricate ^103^Pd brachytherapy seeds by directly depositing ^103^Pd onto carbon bar substrates, thereby eliminating the metallic pre-coatings and the complex pellet assemblies required from prior reports. Under hydrazine-based bath conditions optimized in Li et al. (106), this method achieves a 98% deposition efficiency and a ^103^Pd utilization rate of 51%, which is more than double (∼25%) seen with traditional silver bars (105). By streamlining the plating process and cutting material losses, the approach reduces both fabrication cost and complexity, paving the way for more economical, high-performance ^103^Pd seed production and broader clinical adaptation (105).

**Figure 4:**
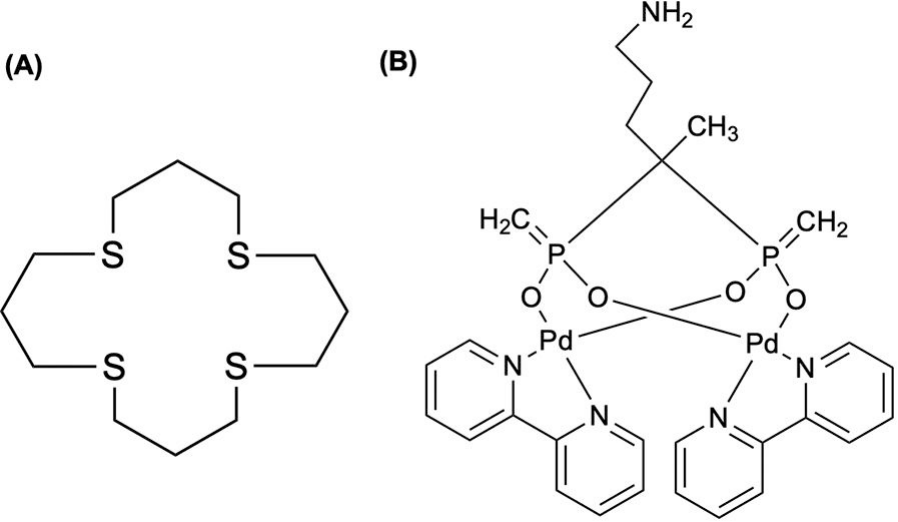
**(A)** A tetradentate thioether macrocycle, 1,5,9,13-tetrathiacyclohexadecane ([16]aneS_4_), has been a suitable chelator for binding Pt(II) and Pd(II) complexes. The chelator has been used to immobilize ^103^Pd in a ^103m^Rh generator (118), where Sporer et al. (111) coupled [16]aneS_4_ to sucrose septaisobutyrate (SSIB) moiety, furnishing a ligand capable of efficiently trapping ^103^Pd within the lactose octaisobutyrate (LOIB) seed. **(B)** The elemental palladium complex with bipyridyl and alendronate ligands, Pd_2_(bpy)_2_ale, was radiochemically synthesized with ^103^Pd and ^109^Pd for radionuclide therapy of bone metastatic tumor cells (115).

Researchers have also explored ^103^Pd in nanoparticle-based brachytherapy. Laprise-Pelletier et al. (107) evaluated the therapeutic efficacy, biodistribution, and tolerability of two formulations of ^103^Pd-doped Pd@Au nanoparticles (NPs) in a prostate cancer xenograft model. Like Djoumessi et al. (108), the Pd NP synthesis achieved a high encapsulation efficiency of 87% for all ^103^Pd atoms incorporated into the 10–14 nm cores (107). Comparing to Moeendarbari et al. (109), who reported 80% tumor inhibition after a 1.5 mCi implant given in 40 μl, the present study achieved similar therapeutic effects using a tenfold smaller volume (4 μl at 1.6–1.7 mCi) (107). Fach et al. (110) formulated ^103^Pd within gold-palladium (AuPd) alloy nanoparticles, intrinsically radiolabeled with ^103^Pd, capable of forming biodegradable gel-like implants upon injection. Therapeutic efficacy of ^103^Pd-nanogels in a tumor-bearing mouse model indicated doses of 25 MBq [^103^Pd]AuPd-nanogel produced a robust tumor-growth delay and double median survival compared to controls, with no systemic toxicity (110). Building on this, Sporer et al. (111) compared injectable ^103^Pd-brachytherapy seeds that form biodegradable LOIB-based solids *in situ*, using either intrinsically radiolabeled PdAuNPs or a novel SSIB-[16]aneS_4_ chelator. The [^103^Pd]PdAuNPs were synthesized by co-reduction of [^103^Pd]PdH_2_Cl_4_ and AuHCl_4_ surface-functionalized with a lipophilic coating and dispersed in LOIB:EtOH to achieve an overall radiochemical yield of 83% or via conjugation of the [16]aneS_4_ chelator shown in Figure 4A to a lipophilic sucrose septaisobutyrate (SSIB), followed by complexation with [^103^Pd]PdH_2_Cl_4_ in 99% yield (111). While both formulations reached activities of 1–1.5 GBq/ml with negligible release (<1%) of radioactivity over 30 days, the chelator strategy deems to be favorable as it avoids non-degradable gold and offers a versatile platform for other radiometals (111).

Hindie et al. (112) used the Monte Carlo track-structure code CELLDOSE (113) (for electrons) in conjunction with PHITS (114) (for photons) to quantify energy deposition from ^103^Pd/^103m^Rh at the cell surface, within the cytoplasm, and in the nucleus enabling normalized comparison against ^161^Tb and ^177^Lu. In the single-cell model, ^103^Pd delivered 7- to 10-fold higher nuclear absorbed dose and 9- to 25-fold higher membrane dose than ^177^Lu—driven primarily by Auger and conversion electrons—with ^161^Tb showing intermediate dose profiles (112). Annamalaisamy et al. (115) reported the first radiosynthesis and evaluation of ^103^Pd_2_(bpy)_2_ale (Figure 4B), designed as an *in vivo* ^103^Pd/^103m^Rh generator for bone-targeted Auger-electron therapy—extending prior work by Cipriani et al. (116) and Fathy et al. (117). At pH of 7 and 60°C, the radiosynthesis achieved >85% radiochemical yield by iTLC, and preparative HPLC confirmed radioactive and non-radioactive complexes were identical (115). Notably, iTLC showed complete retention of parent ^103^Pd and daughter ^103m^Rh—significantly improving upon macrocyclic ^103^Pd/^103m^Rh generators reported by Jensen et al. (118), which exhibited ∼7% ^103m^Rh release—related to the electron-donating bipyridyl ligand quenching “Coulomb explosion” effect discussed in Nath et al. (115, 119). The result from the work of Jensen et al. (118) can be explained by works of van Rooyen et al. (120) and Szucs et al. (121) who conducted detailed recoil energy calculations associated with the emission of Auger electrons, photons, and neutrinos (115). Finally, ^103^Pd_2_(bpy)_2_ale exhibited potent multimodal toxicity via Auger electrons and demonstration chemotoxicity comparable to cisplatin by works of Zhao et al. (122), highlighting its theragnostic potential (115).

### 3.2 Palladium-109, ^109^Pd

^109^Pd (t_1/2_ = 13.7 h) possesses favorable nuclear characteristics suitable for targeted radionuclide therapy and SPECT imaging as it decays by *β*^−^ emission (*E_β_*_(max)_ = 1.12 MeV, 100%) to ^109m^Ag (t_1/2_ = 39.6 s), which then emits an 88 keV photon (*I_γ_* = 3.6%) before it finally decays to ^109^Ag, from a cascade emission of both conversion and Auger electrons (123). As described in Boros and Packard (78), the radionuclide was originally proposed for radiolabeling of antibodies for antitumor therapeutic purposes, but the focus has changed to exploring ^109^Pd-porphyrin complexes as photosensitizing agents for photodynamic therapy of cancer (78). Fawwaz et al. (124) first demonstrated the anticancer capabilities of ^109^Pd by labeling hematoporphyrin and protoporphyrin for controlling homograft rejection (125).

#### 3.2.1 Production and radiochemical separation of ^109^Pd

Highlighted in Table 2, ^109^Pd is produced using an enriched ^108^Pd (98%) metal target, which was performed by Chakraborty et al*.* (123), obtaining a specific activity of ∼1.85 GBq/mg (50 mCi/mg) at a thermal neutron flux of 3 × 10^13^ n cm^−2^ s^−1^ for 3 days (78). In the review by Boros and Packard (78), a dissolution method is carried out in heated aqua regia and is subsequently evaporated and heated to dryness with 12 N HCl to form H_2_PdCl_4_. Silver-111 (^111^Ag) is co-produced and can be removed by coprecipitation with small amount of AgNO_3_ (78). The supernatant containing ^109^Pd is later dissolved in dimethylsulfoxide (DMSO) to produce ^109^Pd(DMSO)_2_Cl_2_ for subsequent syntheses (78). Hien et al. (126) reported thermal neutron capture cross-section (*σ*_0_) and resonance integral (I_0_) of the ^108^Pd(n,γ)^109^Pd, backing previous work of thermal neutron capture cross sections (127–134) and resonance integral data (131, 135) for this reaction.

#### 3.2.2 Applications of ^109^Pd

Porphyrin derivatives are well known to preferentially accumulate in malignant tumors via photodynamic mechanisms (136–139), and early efforts to radiolabel these macrocycles with therapeutic radionuclides—such as ^109^Pd-hematoporphyrin (140) and ^109^Pd-antimelanoma antibodies (141)—demonstrated targeting potential but lacked tumor retention. To expand upon this potential, Das et al. (142) radiolabeled a porphyrin derivative (DHBEP) with n.c.a. ^109^Pd to create a highly stable, rapidly tumor-localizing radiopharmaceutical. The novel ligand DHBEP was synthesized via a two-step sequence and complexed with ^109^PdDMSO_2_Cl_2_ at 80°C for 1 h, achieving >98% radiochemical purity. The ^109^Pd-DHBEP complex remained stable at >97% after 48 h (∼4 half-lives of ^109^Pd) at room temperature in saline (142). Biodistributions studies with Swiss mice bearing fibrosarcoma tumors revealed high tumor uptake at 30 min p.i. [(5.28 ± 1.46%IA/g)] and activity was cleared via the renal pathway (142).

**Figure 5:**
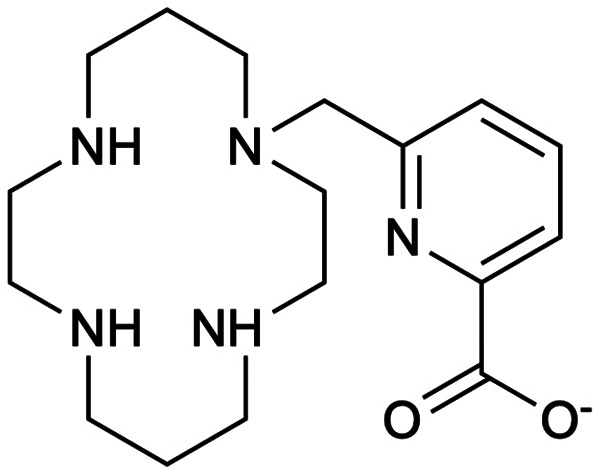
A monopicolinate cyclam, TE1PA, was developed by (255–258) as it exhibited improved properties for ^64^Cu-immuno-PET imaging in terms of radiolabeling yield, conjugation to those of DOTA and NOTA derivatives (125). Pineau et al. (125) investigated the coordination of the chelator with elemental Pd and ^109^Pd to assess the potential development of theragnostic pairs of either ^64^Cu/^103^Pd or ^64^Cu/^109^Pd.

Pineau et al. (125) evaluated TE1PA, shown in Figure 5, to demonstrate its suitability for complexation with both natural and radioactive palladium towards radiopharmaceutical development. Under all conditions and comparing TE1PA to cyclam, TE1Bn (benzyl cyclam), TE1Py (pyridylmethyl cyclam), they reported significant improvement in inertness of [^109^Pd][Pd(TE1PA)]^+^ over [^109^Pd][Pd(cyclam)]^2+^ at room temperature over a 24-h period, highlighting the enhances properties of the picolinate derivative (125).

Gharibkandi et al. (143) developed ^109^Pd-coated gold nanoparticles (Au@^109^PdNPs) functionalized with polyethylene glycol (PEG) conjugated to trastuzumab for targeted therapy of HER2-positive cancers. The resulting Au@Pd-PEG-trastuzumab radiobioconjugate averaged 9.5 antibodies per nanoparticle and demonstrated high HER2-specific uptake in SKOV-3 cells, achieving >99% internalization within 1 h, consistent with findings reported by Gaweda et al. (143, 144). The authors compared the cytotoxicity of radiobioconjugates labeled with the Auger emitter ^125^I (t_1/2_ = 59.49 d; Au@Pd^125^I-trastuzumab), *β*^−^ emitter ^198^Au (t_1/2_ = 2.69 d; ^198^Au-trastuzumab), and the ^109^Pd/^109m^Ag *in vivo* generator (Au@^109^Pd-trastuzumab) (143). With consistent activity concentrations of 20 MBq/ml, the ^109^Pd/^109m^Ag-based conjugate demonstrated significantly higher cytotoxicity than those conjugates radiolabeled with either ^125^I or ^198^Au, highlighting the therapeutic advantage of simultaneous emission of both radiation types from this generator design (143). A subsequent study in 2024 (145) improved ^109^Pd production using ^108^Pd, achieving >500 MBq/mg from the natural palladium target and >2 GBq/mg from the enriched palladium target (78). Their findings indicated that Pd NPs labeled with ^109^Pd were significantly more cytotoxic at similar activities than those labeled with either ^131^I or ^125^I (145). Analogous to ^103^Pd, Annamalaisamy et al. (115) also reported the radiosynthesis and evaluation of ^109^Pd/^109m^Ag *in situ* generator bound to a mixed bipyridyl-bisphosphonate scaffold, ^109^Pd_2_(bpy)_2_ale, for bone-targeted radionuclide therapy. *in vitro*, the conjugate significantly reduced metabolic viability in prostate and ovarian cancer cells, with cytotoxicity depending on both activity concentration and exposure time (115).

## 4 Osmium and osmium-based radionuclide

Naturally occurring osmium consists of seven stable isotopes: ^184^Os (0.02%), ^186^Os (1.59%), ^187^Os (1.97%), ^188^Os (13.24%), ^189^Os (16.15%), ^190^Os (26.26%), and ^192^Os (40.78%) (19). Radioisotopes include ^185^Os, ^191^Os, ^193^Os, and ^194^Os, in this review we will only discuss ^191^Os.

### 4.1 Osmium-191, ^191^Os

^191^Os (t_1/2_ = 15.4 d) decays to ^191m^Ir (t_1/2_ = 4.96 s) by *β*^−^ emission (100%), suitable for an ^191^Os/^191m^Ir generator used for first-pass radionuclide angiocardiography (146). Cheng et al. (147) first used ^191^Os in the development of the ^191^Os/^191m^Ir generator (148). The long half-life facilitates its use in generator construction, quality-control, and clinical use distant from production facilities (146, 149).

#### 4.1.1 Production and radiochemical separation of ^191^Os

Shown in Table 3, Salek et al. (149) irradiated isotopically enriched osmium (^190^Os, 97.8%) in the 5 MW Tehran Research Reactor (ϕ = 4 × 10^13^ n cm^−2^ s^−1^) for 15 days with subsequent fusion in a mixture of KOH-KNO_3_, reporting a specific activity of ∼325 mCi/mg. The dissolution method for osmium reported by Brihaye et al. (150) has been established in subsequent steps to form K_2_OsCl_6_; and carried out for all reported studies in this review. Additionally, osmium by-products ^185^Os (t_1/2_ = 15.4 d) via ^184^Os(n,γ)^185^Os reaction and ^193^Os (t_1/2_ = 30.2 h) via ^192^Os(n,γ)^193^Os reaction are produced only in trace amounts (149). These are neglected as ^185^Os decays to stable ^185^Re, and ^193^Os decays quickly (149). However, an unavoidable longer-lived impurity is ^192^Ir (t_1/2_ = 73.8 d), which is produced when stable ^191^Ir—the stable decay product of ^191^Os—undergoes a ^191^Ir(n,γ)^192^Ir reaction during irradiation (146, 149, 151). Brihaye et al. (150) demonstrated two separation methods—distillation and solvent extraction—between ^191^Os and ^192^Ir. Using these methods, they achieved a separation efficiency of 100% by distillation and 99.9% efficiency by solvent extraction (150). Salek et al. (149) modified the extraction method and yielded a 98.8 ± 0.48% ^191^Os recovery, while completing the procedure in 30 min.

**Table 3:** Production route for ^191^Os.

Radionuclide	Nuclear reaction	Flux/Energy	References
^191^Os	^190^Os(n,γ)^191^Os	4 × 10^13^ n cm^−2^ s^−1^	(149)

#### 4.1.2 Applications of ^191^Os

**Figure 6:**
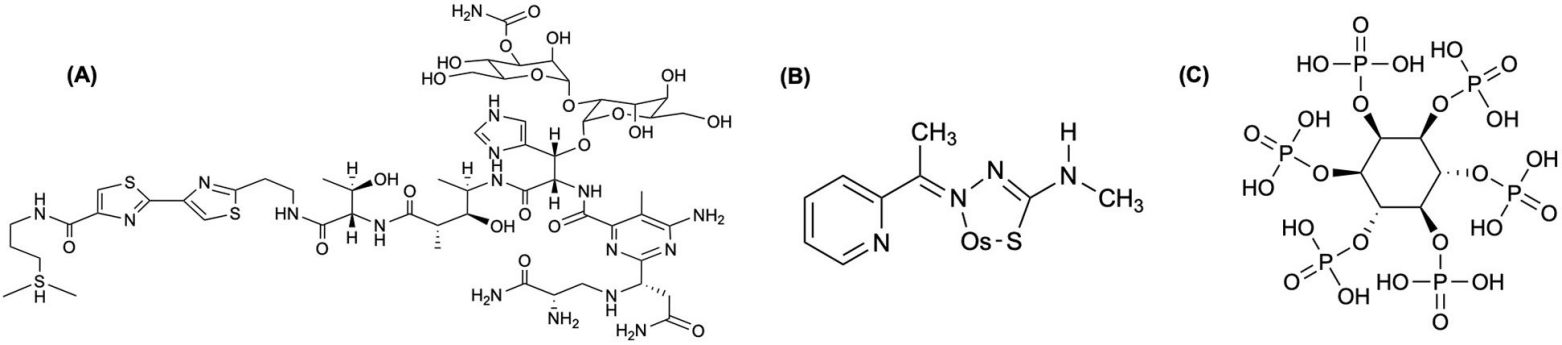
**(A)** Bleomycins (BLMs) are tumor seeking antibiotics that have been widely used in cancer chemotherapy, where these compounds are activated by cation insertion as anti-neoplastic agents; therefore, resulting in DNA decomposition (148). **(B)** Moghaddam et al. (152) labeled 2-acetyl pyridine 4-N-methylthiosemicarbazone (APMTS) with ^191^Os (elemental Os shown in structure) to develop a potential *in vivo* tumor-targeting radionuclide generator. **(C)** Moghaddam-Banaem et al. (151) labeled the salt form of phytic acid, phytate, and radiolabeled with ^191^Os (^191^Os-phytate) to develop an *in vivo* radionuclide generator.

In a study performed by Jamre et al. (148), BLM (Figure 6A) was radiolabeled with ^191^Os by reacting it with K_2_OsCl_6_. The total labeling and formulation of ^191^Os-BLM took approximately 24 h, resulting a >95% radiochemical yield and >97% radiochemical purity, with <3% free ^191^Os- K_2_OsCl_6_ detected by radio-TLC (148). They reported the ^191^Os-BLM complex remained stable in aqueous solution for ∼72 h. Biodistribution studies (4 h, 24 h, 48 h, 72 h, and 14d p.i.) for ^191^Os-BLM demonstrated high uptake in the lungs and moderate accumulation in the liver and spleen, all remaining >1% ID/g throughout the study (148). *In vivo* imaging at 24, 48, and 72 h confirmed these retention patterns as well (148).

Labeling APMTS (Figure 6B) with ^191^Os, Moghaddam et al. (152) achieved >95% radiochemical yield in a 12 h synthesis with a specific activity of 21.5 GBq/mmol, while the complex remained >95% stable for at least 48 h (152). In the biodistribution studies (4, 24, 48, and 72 h p.i.) using the ^191^Os-APMTS complex, liver uptake and kidney uptake peaked by 48 h (5.2%–6.7% ID/g), while there was low blood, heart, bone retention by 24 h and negligible by 72 h (<0.5% ID/g) (152). A follow-up study by Moghaddam-Banaem et al. (151), demonstrated the preparation of ^191^Os-phyate complex shown in Figure 6C that could be used for radiosynovectomy applications. Using 10 mg of sodium phytate, the complex forms in ∼24 h with a labeling yield >98% detected by radio-chromatography, while remaining stable in an aqueous solution for at least 72 h (151). Biodistribution studies (0.5, 4, 24, 72 h p.i.) showed most of the injected dose remained in the joint with minimal uptake in the kidney, and other organs considered negligible (<0.5% ID/g) (151).

## 5 Iridium and iridium-based radionuclide

Iridium has two naturally occurring stable isotopes, ^191^Ir (37.3%) and ^193^Ir (62.7%) (19). Radioisotopes include ^191m^Ir, ^192m^Ir. In this review we will only discuss ^192^Ir.

### 5.1 Iridium-192, ^192^Ir

The radionuclide ^192^Ir (t_1/2_ = 78.83 d) is an important therapeutic radionuclide, particularly in brachytherapy, due to the favorable nuclear properties including 95% *β*^−^ emission (E*_β_*_−_ = 7 MeV) and 5% electron capture (153). Furthermore, two notable *γ*-ray energies include 316 keV (I*_γ_* = 82.7%) and 468 keV (I*_γ_* = 47.8%) (153). Bertermann and Brix (154) obtained preliminary results for the use of ^192^Ir in high dose rate (HDR) brachytherapy to treat prostate cancer (155, 156).

#### 5.1.1 Production and radiochemical separation of ^192^Ir

Due to its widespread use, ^192^Ir is routinely produced in nuclear reactors via the ^191^Ir(n,γ)^192^Ir reaction, using either Na_2_IrCl_6_ targets—described by Ananthakrishnan (157)—or iridium wire, as applied in clinical settings by Schaeken et al. (153, 158). All production routes are shown in Table 4. Irradiating Na_2_IrCl_6_ under standard conditions —10 mg; ϕ = 1.5 × 10^13^ n cm^−2^ s^−1^; 7 days—can yield 12 GBq of ^192^Ir, with specific activity >185 GBq per gram Ir (157). After irradiation, the targets are dissolved in 10 ml of 0.1 N HCl, yielding radiochemical solutions with concentrations ranging from 74 to 370 MBq/ml and >99% radionuclidic purity (157). This method remains the benchmark for high-activity, high-purity ^192^Ir production for clinical brachytherapy (157). As reactor-produced ^192^Ir is carrier-added, accelerator routes have been explored to produce n.c.a. ^192^Ir with potentially higher specific activity.

**Table 4:** Production routes for ^192^Ir.

Radionuclide	Nuclear reaction	Flux/Energy	References
^ 192^Ir	^191^Ir(n,γ)^192^Ir	1–1.5 × 10^13^ n cm^−2^ s^−1^	(157, 158)
	^192^Os(p,n)^192^Ir	19 → 6 MeV	(153, 155, 160)
	^192^Os(d,2n)^192^Ir	21 → 5 MeV	(164)
	^193^Ir(γ,n)^192^Ir	E_max_ = 40 MeV	(165)

Via the ^192^Os(p,n)^192^Ir reaction, Hilgers et al. (153) measured a peak cross-section of 68 ± 8 mb at 9.1 ± 0.5 MeV, while identifying an optimal production window of 8–16 MeV (∼0.16 MBq/μA-h ^192^Ir). The authors confirmed their experimental data with nuclear model codes [EMPIRE-II (96) and ALICE-IPPE (159)] and pointed out that though a cyclotron approach yields lower activity than those achieve via reactor-based production, the specific activity could be much higher (153). They estimated under realistic irradiation conditions (30 h, ϕ = 3.74 × 10^15^ p/s), projected batch yields could reach ∼5.6 GBq—serving as a complementary approach and broadening access to high specific activity ^192^Ir brachytherapy sources (153). Langille et al. (155) demonstrated that a 12.8 MeV proton beam on naturally abundant, electroplated osmium targets yields ^192^Ir with an average measured cross section of 46.4 ± 6.2 mb, which compared well with literature values of Hilgers et al. (153) and Szelecsenyi et al. (160). Targets underwent oxidative dissolution (H_2_O_2_/HCl) and anion-exchange chromatography on Dowex 1 × 8, with the process delivering an overall radiochemical efficiency of ∼80% and radionuclidic purity of 100% (155). Building on established microwave-assisted syntheses of non-radioactive complexes [(ppy)_2_Ir(μ-Cl)_2_Ir(ppy)_2_] and Ir(ppy)_2_(bpy)—reported earlier by Alam et al. (161), Bura et al. (162), and Wu et al. (163)—the authors performed the first radiosynthesis of an iridium cyclometallation reaction by adding n.c.a. [IrCl_6_]^3−^ to the microwave reaction (155). They achieved up to 68% radiochemical purity of Ir(ppy)_2_(bpy) with a maximum specific activity of 0.54 ± 0.14 Ci μmol^−1^ (20 ± 5.2 GBq μmol^−1^) (155).

Tarkanyi et al. (164) reported the first experimental cross sections for the ^192^Os(d,2n)^192^Ir reactions up to 21 MeV, employing a stacked-foil technique with 84.5% enriched ^192^Os targets electrodeposited on 25 μm thick Ni foils, thereby observing a cross sectional peak of 370 ± 46 mb at 12.1 ± 0.8 MeV. Although reactor-based ^192^Ir production yields remain higher, the deuteron route results in a n.c.a. product of ^192^Ir with significantly higher specific activity (153, 164). Compared with the earlier ^192^Os(p,n) process via Hilgers et al. (153), the (d,2n) channel delivers higher cross sections and thick-target yields in the same energy window; however, due to smaller and higher-current proton cyclotrons being more readily available, the choice for the ^192^Os(p,n)-reaction is preferred (164). Dovbnya et al. (165) reported the first experimental demonstration of photonuclear ^193^Ir(γ,n)^192^Ir on natural iridium using a tantalum bremsstrahlung converter integrated within a neutron moderator, which enhanced ^192^Ir yields by ∼50% via the ^191^Ir(n,γ)^192^Ir reaction and delivered up to ∼900 MBq/h under 40 MeV, 4 μA beam conditions. Computational simulations with PENELOPE-2008 software (166)—supplemented by evaluated photonuclear cross sections—accurately reproduced experimental yields for ^192^Ir as well as co-produces isotopes (^190^Ir, ^90^Mo, ^99^Mo), validating the mixed γ- and n-flux model (165). Compared to traditional reactor-based ^191^Ir(n,γ)^192^Ir production (74 MBq/h; >1,000 MBq/h-g) and cyclotron-based ^192^Os(p,n)^192^Ir production (>185 MBq/h; without carrier), this electron-accelerator approach offers competitive batch yields and modular flexibility (153, 157, 165). While the specific activity is low, the authors suggest that optimizing activation-cooling regimes and employing enriched ^193^Ir targets could enable scalable, reactor-free ^192^Ir production suitable for medical and industrial applications (165).

#### 5.1.2 Applications of ^192^Ir

^192^Ir has been significantly utilized in high dose rate (HDR) brachytherapy, offering a steep dose gradient that concentrates therapeutic radiation within tumors while minimizing damage to the surrounding normal tissue (167). Jayakody et al. (167) reviewed a suite of independent verification methods—including radiochromic films, ionization-chamber arrays, plastic scintillation detectors, and TLD/OSLD systems—that have been benchmarked against TPS-calculated dose maps for ^192^Ir. Roussakis and Anagnostopoulos (168) wrote a mini-review on the aspects of the Iridium-Knife, detailing the key physical properties of the ^192^Ir HDR source and illustrating how these underlie its characteristic steep dose gradients.

Nohara et al. (169) reported that 166 localized prostate cancer patients treated with a 44 Gy EBRT and 3 × 6 Gy ^192^Ir HDR boost achieved a 5-year biochemical recurrence-free survival of 93.0%. Shigehara et al. (170) observed a 4-year overall survival of 87.2% and PSA progression-free survival of 82.6% in 84 prostate patients receiving 18 Gy ^192^Ir HDR and 44 Gy EBRT. Chin et al. (171) treated 65 prostate cancer patients with EBRT plus two 8.5 Gy ^192^Ir HDR fractions, reporting a 3-year biochemical disease-free rate of 90.8%. Potter et al. (172) used CT-planned ^192^Ir HDR and 48.6–50 Gy EBRT in 189 cervical cancer patients, achieving 3-year pelvic control of 77.6% and disease-specific survival of 68.6%. Ott et al. (173) demonstrated that interstitial ^192^Ir accelerated partial breast irradiation (APBI) in 69 early-stage breast cancer patients which yielded 100% 2-year local control, minimal acute and late toxicity, in 90% of cases.

Abtahi et al. (174) conducted a systematic review (1984–2020) between ^192^Ir and ^60^Co in GYN cancers. They reported that the 5-year overall survival (OS), local control, disease-free survival (DFS) and high-grade GI/GU toxicity were statistically equivalent between the two (174). Wen et al. (175) compared miniaturized HDR sources for cervical brachytherapy and found nearly identical dose distributions within 25 mm of the source, with equivalent clinical outcomes and toxicity rates. Strohmaier and Zwierzchowski (176) reviewed the physical and logistical aspects of ^60^Co vs. ^192^Ir, concluding that the two radionuclides matched in radial dose function, while delivering equivalent clinical efficacy. Tantivantana and Rongsriyam (177) performed a retrospective analysis of 480 stage IB2-IIIB cervical cancer patients treated between 2004 and 2014, comparing outcomes following HDR brachytherapy with ^192^Ir (274 patients; 57.1%) or ^60^Co sources (206 patients; 42.9%). The study found no statistically significant differences in OS, recurrence rate, or DFS between the ^192^Ir and ^60^Co cohorts (177).

## 6 Rhodium and Rhodium-based radionuclides

Rhodium has one naturally occurring stable isotope, ^103^Rh (100%) (19). Radioisotopes include ^99^Rh, ^101^Rh, ^101m^Rh, ^102^Rh, ^102m^Rh, ^103m^Rh, and ^105^Rh, in this review we will only discuss ^103m^Rh and ^105^Rh.

### 6.1 Rhodium-103 m, ^103m^Rh

An isomer of rhodium that has seen applications in targeted radionuclide therapy due to its Auger electrons is ^103m^Rh (t_1/2_ = 56.1 min). It has also been involved in convenient generator pairs with ^103^Pd and ^103^Ru, respectively, *in vivo* (118, 178).

#### 6.1.1 Production and radiochemical separation of ^103m^Rh

The production for ^103m^Rh is shown in Table 5. Epperson et al. (179) introduced a rapid, high-yield generator for ^103m^Rh by solvent-solvent extraction of RuO_4_ into CCl_4_ achieving 94 ± 0.6% ^103m^Rh yield with 3.8 ± 0.7% ^103^Ru contamination in a single, 15-min extraction. This method contrasts with earlier ion-exchange and distillation approaches referenced by the authors, offering a practical foundation for routine on-demand ^103m^Rh availability (179). Bartos et al. (178) similarly used reactor-produced ^103^Ru (from natural ruthenium irradiation of 36 h, yielding 466 MBq) and separated ^103m^Rh from RuO_4_ extraction. This work laid the foundation for supplying short-lived ^103m^Rh in sufficient quantities for further studies (178). Thery et al. (180) reported the recent progress in ruthenium chemistry for the ^103^Ru/^103m^Rh generator for Auger therapy, describing the main limiting factor being an effective separation between the two radionuclides due to the unpredictable, misunderstood chemistry. Their work overcame prior barriers in earlier solvent-extraction and speciation studies, establishing optimal conditions for examining the experimental feasibility of the generator in the future (180).

**Table 5:** Production routes for rhodium-based radionuclides.

Radionuclide	Nuclear reaction	Flux/Energy	References
^103m^Rh	^102^Ru(n,γ)^103^Ru → ^103m^Rh	3 × 10^14^ n cm^−2^ s^−1^	(178, 179, 184)
	^102^Pd(n,γ)^103^Pd → ^103m^Rh	1.2–1.4 × 10^15^ n cm^−2^ s^−1^	(118, 181, 184)
	^103^Rh(p,n)^103^Pd → ^103m^Rh	E*p* = 14–18 MeV	(118, 181, 184)
^105^Rh	^104^Ru(n,γ)^105^Ru → ^105^Rh	3–8 × 10^13^ n cm^−2^ s^−1^	(188, 191)
	^106^Pd(γ,p)^105^Ru → ^105^Rh	E_max_ = 55 MeV	(195, 197)
	^nat^Pd(p,x)^105^Rh	40 → 4 MeV	(198)

More recently, Jensen et al. (118) demonstrated a solid-phase ^103^Pd/^103m^Rh generator using neutron-activated ^102^Pd targets. They chelated carrier-added ^103^Pd with a lipophilic macrocycle, 16aneS_4_, and loaded it on a C18 cartridge (118). The optimal elution performance for ^103m^Rh was achieved with 1.0 M HCl, yielding a radiochemical purity of 99%, an apparent molar activity of 26.6 MBq/nmol, and an elution yield of 5.81% (118). Despite the potential, the low elution yield indicates that further optimization is necessary to utilize the generator for extended use, particularly in clinical applications (118). Ohya et al. (181) improved on this by testing various anion-exchange resins—inspired by Berk (182) and Mamadaliev et al. (183)—following a separation method described in Ohya et al. (101). Four commercially available gel-type anion-exchange resins with comparable functions groups and matrixes were investigated: IRA410 and SA20A (dimethylethanol ammonium), and IRA904 and SA11AL (trimethyl ammonium) (181). Of these, SA11AL delivered the best performance, with a raw yield of 39% and lowest ^103^Pd breakthrough of 0.29% over 32 milking cycles spanning eight weeks (181). More recently, Zagryadsky et al. (184) performed measurements of the ^102^Pd(n,γ)^103^Pd and ^102^Ru(n,γ)^103^Ru reactions in the IR-8 Reactor for the purpose of ^103^Ru/^103m^Rh and ^103^Pd/^103m^Rh generators. They indicated the experimental channel of the IR-8 reactor will be capable of achieving sufficiently ^103^Ru and ^103^Pd for the utilization of ^103m^Rh in radiopharmaceuticals (184).

#### 6.1.2 Applications of ^103m^Rh

Bernhardt et al. (185) performed Monte Carlo simulations to model the metastatic growth of tumor sizes for radionuclide therapy, comparing between high-energy electron emitter ^90^Y (t_1/2_ = 64.05 h), medium-energy electron emitter ^177^Lu (t_1/2_ = 6.65 d), and the low-energy electron emitter ^103m^Rh. They observed for low tumor-to-normal (TNC) tissue activity concentrations, ^103m^Rh performed slightly better compared to ^177^Lu; however, for high TNC values, ^103m^Rh was the best choice for tumor treatment (185). However, as the authors noted, the short half-life (t_1/2_ = 56.1 min) may be a limitation in the adaptation as an optimal radiotherapeutic (185).

### 6.2 Rhodium-105, ^105^Rh

^105^Rh (t_1/2_ = 35.36 h) is an attractive candidate for radiotherapeutic applications due to its nuclear characteristics (186). ^105^Rh decays via *β*^−^-emission with energies of 179 keV (75.0%), 74 keV (5.2%), and 70 keV (19.7%), along with two low-abundant *γ*-rays at 319 keV (*I_γ_* = 20%) and 306 keV (I*_γ_* = 5%)—useful for mapping the *in vivo* uptake of the administrator radiopharmaceutical (78, 187, 188). Grazman and Troutner (189) first explored the viability of ^105^Rh and its properties for use as a radiotherapeutic agent (190).

#### 6.2.1 Production and radiochemical separation of ^105^Rh

**Figure 7:**
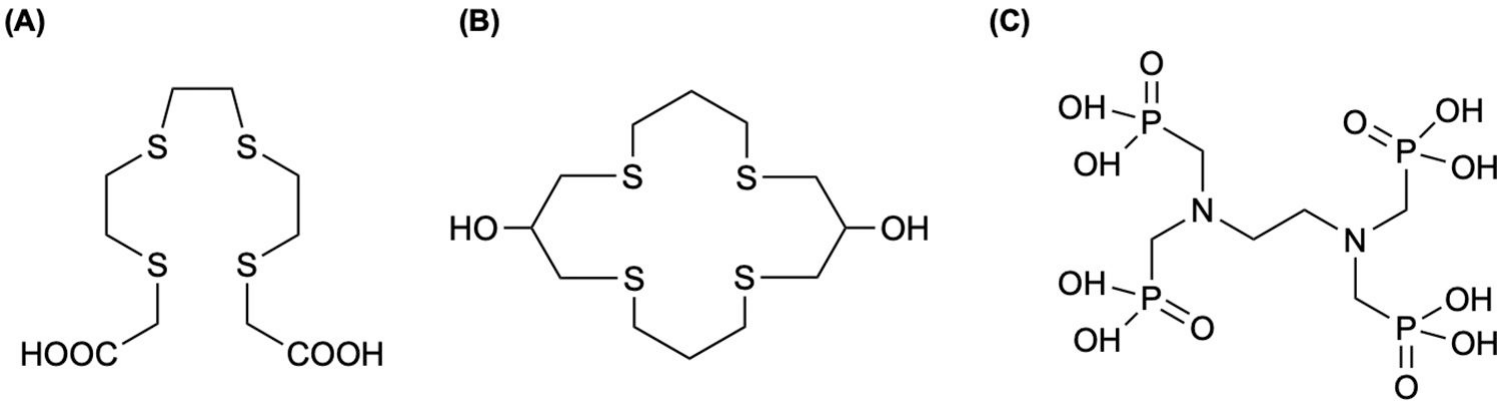
Okoye et al. (192) labeled ^105^Rh successfully with previously used chelators for Rh(III) complexation, **(A)** the tetrathioether ligand (222-S_4_-diAcOH) by Goswami et al. (193) and **(B)** the tetradentate thiamacrocyclic ligand (16S_4_-diol) by Venkatesh et al. (207). **(C)** In an early study by Ando et al. (259), ^177^Lu can be chelated to ethylenediamine-tetra-methylene phosphonic acid, EDTMP, producing a bone-seeking phosphonate complex that is chemical and biologically stable. Therefore, the same group (199) investigated the biological behavior of ^105^Rh when chelated to EDTMP.

Described in Table 5, Jia et al. (191) developed a scalable route to n.c.a. ^105^Rh by irradiating enriched ^104^Ru in the MURR reactor (*ϕ* = 8 × 10^13^ n cm^−2^ s^−1^, 72 h), achieving average yields of ∼5 mCi per mg Ru and >85% total recovery of ^105^Rh. Their MgO adsorption method eliminated the need for chlorine gas and the formation of RuO_4_—required in an earlier approach (189)—while delivering a ruthenium decontamination factor of 16,600, supporting the reliable availability of ^105^Rh in large quantities (191). Subsequently, Unni et al. (188) developed a methodology for the production and purification of carrier-free ^105^Rh by irradiating natural Ru (99.9%) at a thermal flux of 3 × 10^13^ n cm^−2^ s^−1^ for 5–7 days, followed by a 24 h decay of ^105^Ru to ^105^Rh, achieving within 5% of ≈24 mCi predicted by Bateman's equation. The authors oxidized the Ru matrix (^97^Ru, ^103^Ru, and trace ^192^Ir) to volatile RuO_4_ (KIO_4_/KOH at 70°C, 20 min), performed successive solvent extractions with CCl_4_ (retaining 97.8 ± 0.78% of ^105^Rh in aqueous phase), and then applied 100% TBP extraction to obtain 95.35 ± 0.78% of ^105^Rh (aqueous phase) and 96.6 ± 0.8% of ^192^Ir (organic phase) (188). A co-precipitation of ^105^Rh with Fe(III) as hydroxide using KOH recovered 89.4 ± 2.2% of ^105^Rh, and a three-stage Fe removal—using cationic exchange chromatography—delivered a final overall recovery of ∼80% (15–20 mCi) of carrier-free ^105^Rh (188). Okoye et al. (192) demonstrated a comprehensive strategy to reclaim, purify, and reuse enriched ^104^Ru targets—originally captured as RuO_4_ in 3 M HCl from decades of ^105^Rh production—for economical, high-yield ^105^Rh manufacture. The recycled metal retained 98.84% ^104^Ru enrichment—a slight decrease from their original—and enabled up to 97.3% ^105^Rh recovery (19.10 mCi) (192). The isolated ^105^Rh was subsequently used in radiolabeling experiments with two previously developed chelators (193, 194), yielding radiochemical efficiencies of 91.0% ± 1.5 for 222-S_4_-diAcOH (Figure 7A) and 80.9% ± 0.4 for 16S_4_-diol (Figure 7B) (192).

Inagaki et al. (195) investigated the production of ^105^Rh via two distinct routes: neutron irradiation of ^nat^RuO_4_ powder (*ϕ* = 4.5 × 10^12^ n cm^−2^ s^−1^, 10 min) through the ^104^Ru(n,γ)^105^Ru reaction; and bremsstrahlung photon irradiation of natural Pd foils (5 × 5 mm^2^) at 20–40 MeV using an electron linear accelerator (linac), inducing the ^106^Pd(γ,p)^105^Ru reaction. To enable comparison, the authors normalized yield data to equivalent target masses, beam currents, and irradiation times, reporting 77 ± 2 kBq of ^105^Rh via the reactor method (10 mg) and 88 ± 5 kBq at 40 MeV from the linac method (50 mg, 100 μA, 10 min) (195). Furthermore, extrapolation to clinical-scale conditions using the linac method—10 g Pd target, 1 mA current, and 24 h irradiation—predicted a ^105^Rh yield of approximately 20.1 GBq, far exceeding the 0.148 GBq typically required for diagnostic or therapeutic applications, as described in Sciuto et al. (195, 196). Kazakov et al. (197) investigated a method for producing carrier-free ^105^Rh using a 55 MeV electron accelerator, analyzing the isotopic composition of irradiated PdCl_2_ and optimizing separation methods. Irradiation of 270 mg PdCl_2_ in 5 ml solution at 100 nA for 1 h yielded 73.7 kBq/μAh of total rhodium activity, with ^105^Rh containing 82% (60 kBq/μAh, 2.1 kBq (197). When compared to Inagaki et al. (195), who reported 88 kBq from 50 mg ^nat^Pd foil for at 40 MeV (10 min, 100 nA), both demonstrated feasible accelerator-based alternatives to reactor or cyclotron production for medical applications (197). Nonetheless, the irradiated PdCl_2_ was dissolved in 2 M HCl and passed through extraction chromatography columns using either DGA-Normal or TEVA resins (197). Column and distribution coefficient studies showed DGA-Normal offered superior performance, eluting ≥98% of ^105^Rh in 2 M HCl and enabling complete Pd stripping with 11 M HCl (Pd/Rh separation factor >10^5^), while TEVA failed to achieve sufficient Pd/Rh separation (197).

Khandaker et al. (198) reported the first experimental measurement of ^nat^Pd(p,x)^105^Rh excitation function from 4 to 40 MeV using stacked-foil activation, observing significant discrepancies between measured cross-sections and nuclear model predictions from TALYS (49) and ALICE-IPPE (159). From the experimental data, thick target yield calculations suggest that low-energy cyclotrons (E < 20 MeV) can effectively produce ^105^Rh, primarily via the ^108^Pd(p,α)^105^Rh reaction (198).

#### 6.2.2 Applications of ^105^Rh

Jurisson et al. (187) investigated ^105^Rh radiopharmaceutical development by exploring a suite of cis- and trans-[RhCl_2_l]^+^ complexes using tetradentate thioether ligands. Brooks et al. (190) reported the synthesis and purification of novel ^105^Rh-bleomycin (^105^Rh-BLM) complex, demonstrating >80% complexation yield, high *in vitro* stability, and rapid biphasic *in vivo* clearance with minimal non-specific retention. Although ^105^Rh-BLM achieved tumor uptake approximately four-fold greater than contralateral muscle, its potential for targeted radiotherapy is limited by significant levels and prolonged retention in the kidneys relative to tumor (190). The study by Ando et al. (199) evaluated ^105^Rh as a candidate for radiotherapeutic applications targeting bone metastases by leveraging its favorable decay properties and investigating its biological behavior when chelated to EDTMP shown in Figure 7C. Radiolabeling with EDTMP achieved >99% labeling efficiency, with no dissociation observed for up to 5 days at room temperature (199). Compared to a study using ^99m^Tc-MDP by Sanada et al. (200), ^105^Rh-EDTMP demonstrated comparable bone uptake, but exhibited faster clearance from circulation and significantly higher bone-to-tissue ratios (199). Mentioned in Okoye et al. (192), a variety of chelates have been evaluated (186, 193, 194, 201–213), along with preclinical biological distribution studies have been highlighted in Li et al. (209) and Goswami et al. (193) for ^105^Rh clinical utility towards advancing therapeutic radiopharmaceuticals.

## 7 Ruthenium and ruthenium-based radionuclides

Ruthenium has seven naturally occurring isotopes: ^96^Ru (5.6%), ^98^Ru (1.87%), ^99^Ru (12.76%), ^100^Ru (12.6%), ^101^Ru (17.06%), ^102^Ru (31.55%), and ^104^Ru (18.62%) (19). Radioisotopes of ruthenium include ^97^Ru, ^103^Ru, and ^106^Ru, where our review will focus on ^97^Ru and ^103^Ru. ^106^Ru, which has been predominantly involved in brachytherapy in the last 25 years (4, 214–227), was omitted in this review, due to lack of applications in nuclear medicine.

### 7.1 Ruthenium-97, ^97^Ru

^97^Ru (t_1/2_ = 2.8 d) decays by electron capture (100%) to ^97^Rh, with the emission of low-energy *γ*-rays, 216 keV (86%) and 324 keV (11%) (228). This radionuclide provides excellent conditions for *in vivo* imaging, as it is within the energy window of clinical SPECT detectors (228).

#### 7.1.1 Production and radiochemical separation of ^97^Ru

The production routes for ^97^Ru are listed in Table 6. Zaitseva et al. (229) measured excitation functions for ^97^Ru production via the ^99^Tc(p,3n)^97^Ru reaction, using a stacked-foil technique (50–100 nA) from 20 to 99 MeV. They measured a 438 ± 66 mb peak at 32 MeV—corresponding to a thin-target yield of ∼934 μCi/μAh—and a cumulative yield of ∼10.49 mCi/μAh when degrading protons from 99 MeV to the threshold (E_th_ = 18.3 MeV) (229). An optimal 19–50 MeV window maximized ^97^Ru production (∼7 mCi/μAh) while higher-energy beams (>50 MeV) could push yields beyond 10.5 mCi/μAh for Ci-scale production at higher currents (229). Building on this, Zaitseva et al. (230) optimized a radiochemical separation for metallic Tc targets irradiated at 50 MeV (∼8 μA, 1 h), isolating 40–50 mCi of ^97^Ru. A four-step process—dissolution, acid conversion, oxidation-distillation, and absorption—reduced Ru(VIII) to Ru(III) and recovered 95%–98% of Ru with >10^4^ purity after 6–7 h (230). An estimated delivery of ≥150 mCi of ^97^Ru is needed (50 MeV, 6–8 μA, 8 h) for clinical purposes to be feasible ∼70 h after EOB (230).

**Table 6:** Production routes for ruthenium-based radionuclides.

Radionuclide	Nuclear reaction	Flux/Energy	References
^ 97^Ru	^99^Tc(p,3n)^97^Ru	E_max_ = 99 MeV	(229, 230)
	^nat^Mo(α,n)^97^Ru	E_max_ = 67 MeV	(231, 232, 237, 238)
	^89^Y(^12^C,4n)^97,97m^Rh → ^97^Ru	70 → 65 MeV	(239)
	^89^Y(^12^C,p3n)^97^Ru	70 → 65 MeV	(239)
^ 103^Ru	^nat^Mo(α,n)^103^Ru	40 → 8 MeV	(231, 232)
	^232^Th(p,f)^103^Ru	E_max_ = 89.6 MeV	(245)
	^nat^Ru(n,γ)^103^Ru	5–10 × 10^14^ n cm^−2^ s^−1^	(238, 243, 246)

Ditroi et al. (231) and Tarkanyi et al. (232) explored α-induced routes on natural molybdenum, measuring ^97^Ru excitation functions up to 40 MeV. Both found peaks near 39 MeV (182.4 ± 20.5 mb and 232 ± 26 mb, respectively), along with good agreement from previous results by Levkovskij (233) and Graf and Munzel (234) across all energy ranges, and with Rapp et al. (235) at low energies (231, 232). Model comparisons (TENDL-2011/TENDL-2015 (49), ALICE-IPPE (159), and EMPIRE-3.1 (96)) generally agreed in trend, with (232) calculating thick-target yields reaching 2 GBq/C (0.19 mCi/μAh), and potential to increase the yield by a factor of three through isotopic enrichment favoring the ^94^Mo(α,n), ^95^Mo(α,2n), and ^96^Mo(α,3n) reactions. Thick target yields were described by Abe et al. (236), thereby obtaining a yield of 126 μCi/μAh via the ^94^Mo(α,n) reaction using 30 MeV α-particles (232). Sitarz et al. (237) extended α-induced production of ^97^Ru to 67 MeV, confirming a 237 ± 20 mb at 41.8 MeV, agreeing with (232) and (231) below 40 MeV. Most recently, Happl et al. (238) demonstrated ^97^Ru production for α-induced irradiation of ^nat^Mo for 10 h to yield >300 MBq end of irradiation (EOI). Post-irradiation, the target foil was dissolved and bulk Mo was removed using two sequential ion exchange columns; obtaining trace impurities of Mo (0.9–2.0 μg) and minor radionuclidic contaminants including ^95^Ru (t_1/2_ = 1.6 h), ^95m^Tc (t_1/2_ = 61.0 h), and ^95^Tc (t_1/2_ = 20.0 h) in the ^97^Ru eluate (238). The reported radiochemical yield of ^97^Ru was 40%–56%, resulting in deliverable activities of 87–123 MBq (74–106 MBq/ml) (238).

Furthermore, Maiti and Lahiri (239) introduced a novel ^12^C + ^89^Y production route for n.c.a ^97^Ru, while avoiding co-production of longer-lived radionuclides to achieve tracer-level yields after cooling. Furthermore, the authors developed a two-separation scheme—a solid-liquid extraction in 1 M HCl and sequential 0.1 M/6 M HCl column chromatography—yielding 88% n.c.a. ^97^Ru and resulting distinct Ru(IV)/Ru(III) speciation under certain conditions (239).

#### 7.1.2 Applications of ^97^Ru

Oster et al. (240) evaluated ^97^Ru-DTPA as a potential imaging agent for cerebrospinal fluid by injecting 0.4 mCi of the compound into the cisterna magna of dogs, while comparing the performance with ^111^In-DTPA. From their study, they established ^97^Ru-DTPA to be superior to ^111^In-DTPA as it delivered approximately half the absorbed dose to the tissues, along with better imaging capabilities (240). Som et al. (241) labeled transferrin with ^97^Ru (^97^Ru-TF) and compared its biodistribution to ^67^Ga-citrate, ^123^I-transferrin, ^99m^Tc-plasmin, ^125^I-fibrinogen, and ^131^I-albumin in tumor and abscess bearing animals. Notably, the difference between ^97^Ru-TF and ^67^Ga-citrate were of particular focus, as tumor concentrations of ^97^Ru-TF increased substantially with time, whereas the ^67^Ga concentration did not (241). The authors noted although there were no significant advantages using ^97^Ru over ^67^Ga, the nuclear characteristics of ^97^Ru may improve imaging quality (241).

**Figure 8:**
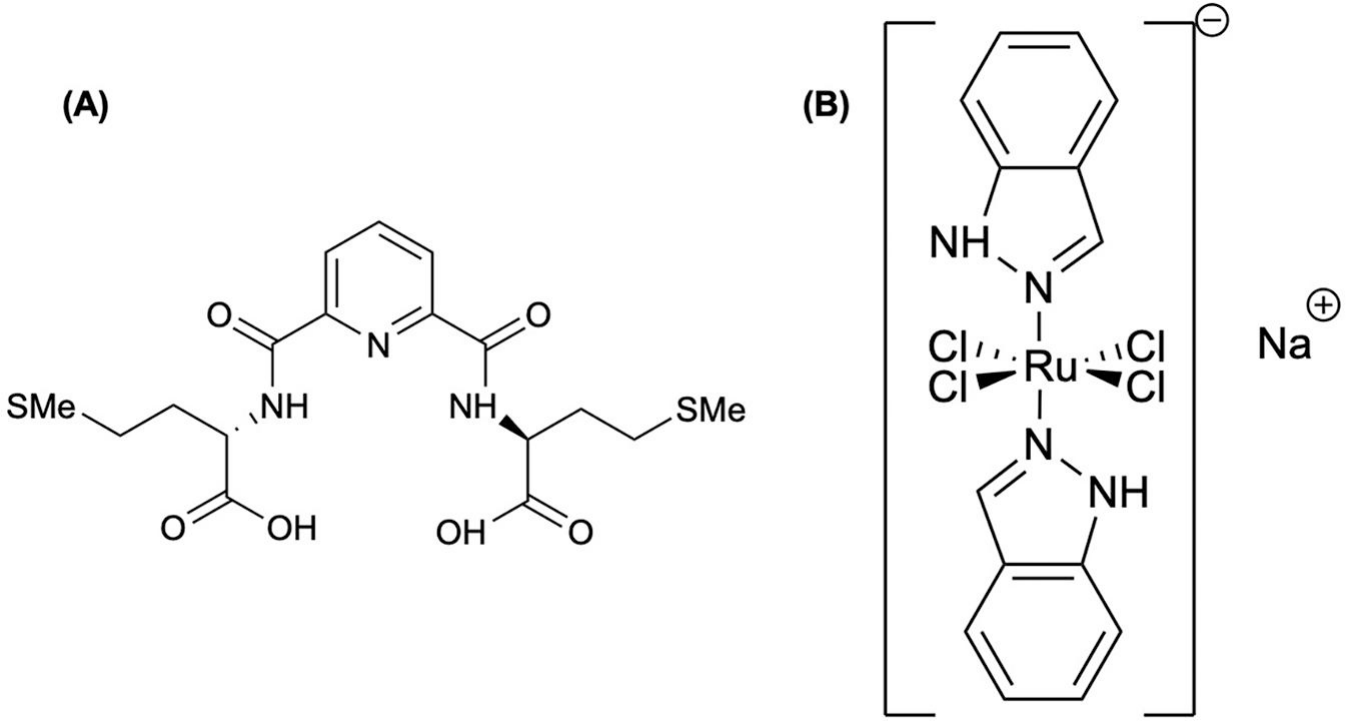
**(A)** Borisova et al. (242) synthesized a peptidomimetic conjugate of natural methionine and pyridine-2,6-dicarboxylate and labeled the ligand with ^97^Ru for potential radiopharmaceutical utilization. **(B)** One of the more promising Ru(III) anticancer complexes is BOLD-100, formerly called IT-139 or KP1339, which is undergoing clinical investigation (238). Although the structure is shown as elemental Ru, Happl et al. (243) radiosynthesized c.a. [^97/103^Ru]BOLD-100 described in this section.

More recently, as a potential radiopharmaceutical, Borisova et al. (242) reported the first ^97^Ru complex with pyridine-2,6-dicarboxamide conjugate shown in Figure 8A (243). Happl et al. (238) further explored the same method from Happl et al. (243) for a three-step synthesis for radiolabeling BOLD-100 (Figure 8B) with c.a. [^97^Ru]RuCl_3_ (0.2–0.5 MBq/μmol). The radiochemical purity of all three intermediates was >99% (238). The final product exhibited an overall radiochemical yield of 8% and an overall chemical yield of 13%, based on the mass of isolated intermediates and products (238). Additionally, the specific activity at the end of synthesis was 0.1 MBq/mg, with a molar activity of 0.05 MBq/μmol (238). Although radiolabeling BOLD-100 with ^97^Ru was successful, the radiochemical yield and specific activity must be improved to enable SPECT imaging using c.a. [^97^Ru]BOLD-100 (238).

### 7.2 Ruthenium-103, ^103^Ru

^103^Ru (t_1/2_ = 39.3 d) decays by β^−^-emission (100%), and has two *γ*-rays, 497 keV (91%) and 610 keV (6%) (238). Although this radionuclide has therapeutic applications, its use in the ^103^Ru/^103m^Rh generator is of importance as well.

#### 7.2.1 Production and radiochemical separation of ^103^Ru

The production routes for obtaining ^103^Ru are highlighted in Table 6. The measured excitation function in Ditroi et al. (231) demonstrated a maximum cross-section of 10.6 ± 1.2 mb at 13.8 ± 0.6 MeV, then gradually declined and plateaued between 18 and 40 MeV, with cross sections ranging from 0.5 to 5 mb (231). The experimental results aligned closely with earlier measurements by Graf and Munzel (234) and Esterlund and Pate (244), though discrepancies in peak values were observed across the studies (231). TENDL-2011 (49) underestimated the experimental cross-sections and exhibited a shift towards the lower energies for the maximum, while EMPIRE-3.1 (96) better replicated the shape of the curve but slightly overestimated the maximum value (231). Integral yield data indicated that α-induced production of ^103^Ru is inefficient—due to its low cross-sections—compared to ^97^Ru (mentioned in 8.1.1), with practical yields falling well below the MBq/μAh range (231). Tarkanyi et al.'s (232) experiment demonstrated a rise in cross section from threshold to a peak of 15.6 ± 1.7 mb at 13.79 ± 0.6 MeV, following a gradual decline and plateau between 18 and 40 MeV, with values ranging from approximately 6.2 to 1.2 mb (232). The authors reported good agreement with the corrected data of Ditroi et al. (231) and earlier measurements by Graf and Munzel (234) and Esterlund and Pate (244), except for a discrepancy by a factor of two near the absolute maximum (232). The TENDL-2011 and TENDL-2015 (49) libraries were found to underpredict the experimental cross sections and shifted the peak position toward lower energies, whereas EMPIRE-3.1 (Rivoli) (96) best reproduced both the shape and magnitude of the experimental excitation curve (232).

Mastren et al. (245) developed a two-step chromatographic purification scheme for obtaining ^103^Ru from proton irradiation on a thorium target. Elution with 30 ml of 10 M HNO_3_ (fractions 8–15) recovered 85 ± 5% of ^103^Ru with a radiochemical purity of 82%, where they reported main impurities of ^117m^Sn and ^125,126^Sb with trace amounts of ^230,233^Pa, ^95^Nb, and ^95^Zr in this fraction (245). To remove those impurities, the DGA resin was incorporated, yielding a final ^103^Ru recovery of 83 ± 5% with a radiochemical purity of >99.9% (245).

Blicharska et al. (246) developed a streamlined separation process to obtain ^103^Ru as a surrogate for fission produced ^106^Ru for the utilization in brachytherapy sources. The authors explored the Ru extraction efficiency in various oxidizing solutions, reporting H_5_IO_6_ to demonstrate the highest conversion of Ru(III/IV) to RuO_4_ with 86.1% extraction (246). The method proved to be sufficiently scalable to produce hundreds GBq of ^106^Ru per liter of PUREX raffinate (246). More recently, Happl et al. (243) obtained [^103^Ru]RuCl_3_• xH_2_O by neutron activation with the Production Neutron Activation (PNA) installation at the spallation neutron source SINQ at Paul Scherrer Institute. The irradiation occurred over a three-week period at a neutron flux of 4 × 10^13^ n cm^−2^ s^−1^ with five ampoules containing 40–50 mg ^nat^RuCl_3_• xH_2_O that were then dissolved in concentrated hydrochloric acid, thereby resulting in activities up to 185 MBq (3.7–4.7 MBq/mg) (243). Happl et al. (238) improved their methods by obtaining ^103^Ru via thermal neutron irradiation (5–10 × 10^14^ n cm^−2^ s^−1^) for 6–8 d using ^nat^Ru metal foils enclosed in quart ampoules, yielding 1,049 MBq at end of irradiation (EOI). From this, c.a.^103^Ru was recovered with radiochemical yields of 81%–82% (up to 648 MBq), molar activities up to 19.4 MBq μmol^−1^ (249 MBq ml^−1^), and a radionuclide purity of >99.9% (238).

#### 7.2.2 Applications of ^103^Ru

**Figure 9:**
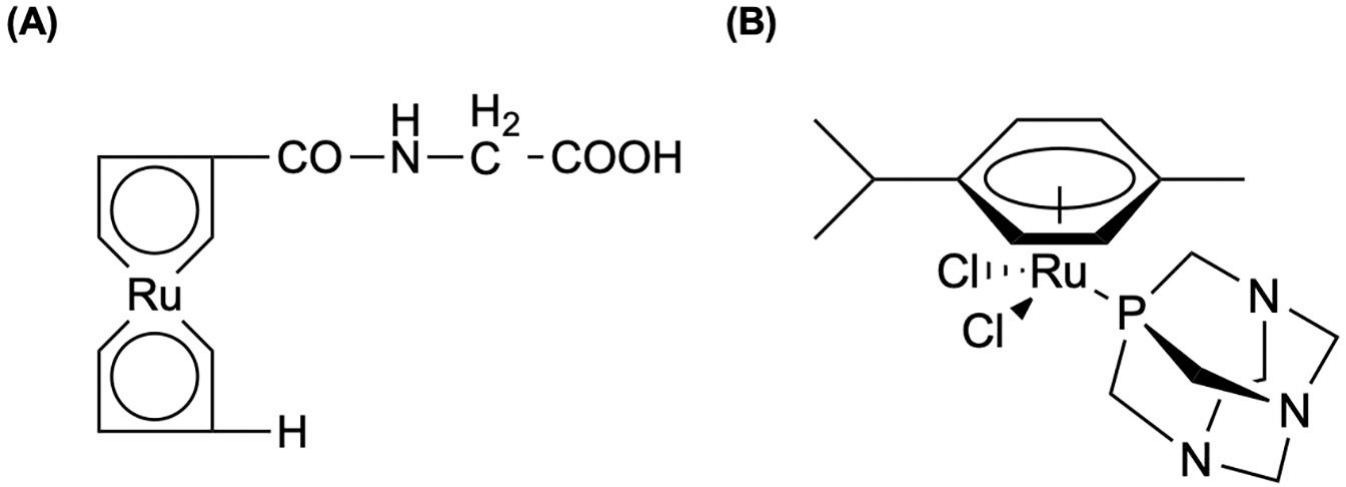
**(A)** Synthesizing a metallocene analog of iodo-labeled (hippuran), ruthenocenoly-glucine (ruppuran), wenzel et al. (248) injected ^97/103^Ru-labeled ruppuran in rabbits. **(B)** Weiss et al. (249) demonstrated the prototype compound, [Ru(*η*^6^-*p*-cymene)Cl_2_(pta)], where pta = 1,3,5-triaza-7-phosphaadamantane (RAPTA-C), reduces the growth of primary tumors in preclinical models for ovarian and colorectal carcinomas while being radiolabeled with ^103^Ru. Both chemical structures show elemental ruthenium.

Tanabe (247) reported ^103^Ru scintigraphy in 37 patients with various types of malignant tumors. In the cohort of four lung cancer patients, ^103^Ru failed to reliably differentiate carcinoma from inflammatory lesions under the study conditions. Wenzel et al. (248) synthesized a metallocene-based analog of iodo-hippuran—ruthenocenoyl-glycine (ruppuran) shown in Figure 9A—and labeled it with ^103^Ru to directly compare its renal clearance kinetics with ^125^I-labeled hippuran. The authors reported similar renal and plasma clearance pattern between the two compounds (248). Moreover, they did report absorbed doses to kidney and bladder with using ^97^Ru-ruppuran as well, achieving slightly lower than that of ^123^I-hippuran, with the results of clearance studies and dose estimates encouraging further kidney scintigraphy and secretory renal function measurements regarding the ^97^Ru-labeled compound (248). Weiss et al. (249) demonstrated radiolabeled [^103^Ru]RAPTA-C (Figure 9B) to be a promising compound for translation to clinical evaluation as it rapidly cleared from the organs and the excreted by the kidneys.

Happl et al. (243) modified a three-step synthesis—published and patented for non-radioactive BOLD-100 in 2018 (250)—of [^103^Ru]BOLD-100 using 1.8–4.2 MBq/mg c.a. [^103^Ru]RuCl_3_, obtaining a >93% radiochemical purity of all three compounds and >38% overall radiochemical yield in the final product. Cytotoxicity of BOLD-100 and [^103^Ru]BOLD-100 were compared in human colon carcinoma (HCT116) and murine colon carcinoma (CT26) cell lines using the colorimetric MTT assay with an exposure time of 96 h (243). The authors reported no effects to the biological activity *in vitro* even at low specific activities of 0.5–1.4 MBq/mg for [^103^Ru]BOLD-100 (243). Furthermore, biodistributions studies with both BOLD-100 and [^103^Ru]BOLD-100 were conducted in Balb/c mice bearing CT26 allografts over a period of 72 h (243). The authors reported from their tissue distribution studies that sub-equimolar amounts of c.a. [^103^Ru]BOLD-100 achieved a higher and prolonged tumor uptake over 72 h, establishing a potential theragnostic approach with ^103^Ru and ^97^Ru once diagnostic SPECT imaging studies with c.a. [^97^Ru]BOLD-100 are performed (238).

## 8 Discussion

In this review, we discussed eleven PGM radionuclides—^191^Pt, ^193m^Pt, ^195m^Pt, ^103^Pd, ^109^Pd, ^103m^Rh, ^105^Rh, ^191^Os, ^192^Ir, ^97^Ru, and ^103^Ru—that offer unique nuclear characteristics involving their half-lives, decay modes, and coordination chemistry suited to both diagnostic imaging and TRT. Across the radionuclides, we address two overarching themes: (1) production and separation challenges or solutions that require high specific activity and radionuclidic purity, and (2) introducing novel chelators and implementing strategies to utilize a specific radionuclide effectively. The optimal production route balances the yield, specific activity, and managing radionuclidic impurities accordingly. Reactor-based methods yield high activities but are often composed of carrier-added products, whereas accelerator routes deliver n.c.a. production—for example, ^191^Pt via ^nat^Ir(p,xn) and ^103^Ru via ^nat^Mo(α,x)—at the expense of enriched targets and complex target dissolution methods. Innovative *in vivo* generator systems, most notably, ^103^Pd/^103m^Rh, ^103^Ru/^103m^Rh, and ^109^Pd/^109m^Ag show promise for implementing short-lived radionuclides; however, must overcome yield limitations relative to established generators clinically: ^88^Ge/^88^Ga, ^44^Ti/^44^Sc, ^62^Zn/^62^Cu, and ^72^Se/^72^As (118, 181, 251). Moreover, emerging nanoparticle-based brachytherapy with ^103^Pd and ^109^Pd and theragnostic applications of ^195m^Pt-labeled complexes highlights the potential for seamless diagnostic-to-therapy transitions without altering compound pharmacokinetics. As future avenues for personalized theragnostics are of importance, platinum-based radionuclides that may offer suitable characteristics but were not mentioned in detail for this review were ^188^Pt (t_1/2_ = 10.2 d), ^189^Pt (t_1/2_ = 10.87 h), and ^197^Pt (t_1/2_ = 0.83 d) (252). Preliminary production and chemical separation methods have been explored regarding these radionuclides, highlighted particularly in Bonardi et al. (21), Neves et al. (253), Smith et al. (252), and Wren et al. (254).

The future for PGM radionuclides may not be mainstream in the clinic; however, the recent research trends towards an optimistic future, especially regarding the radionuclides that were discussed in this review. Over the past decade, there have been notable progressions with differing radionuclides, whether through demonstration of optimal production routes and conditions, innovative separation techniques, or implementation of novel compounds for *in vivo* or *in vitro* studies. However, there are key components that deserve attention. One of the motivations is scaling up production and accessibility, as these radionuclides are produced by not only different target material but also different production pathways which makes this continued development critical. However, from this review, we have seen researchers make progress in optimizing production for certain radionuclides, yielding high specific activities that are feasible for clinical studies. Furthermore, improvements regarding radiochemical separation and recycling methods need to be of focus for cost-effective production and automative radiochemical workflows. We have seen established radiochemical separation techniques carried out to ensure high purity and yields, along with recycling of costly enriched targets to be achievable with minimal loss of enrichment (192). As future work continues to expand upon radiochemistry, the recovery of PGMs post-irradiation, minimizing radioactive waste, and developing automated separation systems may enable sustainability—economically and environmentally—for routine PGM radionuclide use later down the line. A more exciting, future component of PGM radionuclides is integrating them with novel classes of targeting agents—potentially those that have not yet been explored—that would be essential in broadening the toolkit for radiopharmaceuticals. Lastly, the pathway to ensure mainstream adoption of PGM radionuclides will require clinical evidence of safety and efficacy.

To expand upon ^191^Pt's potential, improvements are needed in scaling up production on enriched iridium targets, along with developing automative dissolution/separation techniques to ensure reliable, high specific activity supply. Optimizing irradiation parameters will be required to elevate the small batch yields achieved for ^193m^Pt, while *in vivo* evaluations of ^193m^Pt-labeed complexes should be explored upon their therapeutic efficacy, DNA damage profiles, and off-target toxicity. As Aalbersberg et al. (72) noted, enhanced purification protocols need to be developed for ^195m^Pt, while taking advantage of its unique characteristics may open future opportunities in theragnostics. While nanoparticle-based approaches for ^103^Pd are showing promise, future work must focus on taking the next step from preclinical to clinical studies, while validating dosimetry, biodistribution, and long-term safety. Like ^103^Pd, ^109^Pd-porphyrin and nanoparticle-based approaches require extensive investigation *in vivo* stability, tumor uptake, and scale-up of n.c.a. ^109^Pd production to support clinical studies. To exploit ^103m^Rh's Auger emissions, efforts should focus on improving both ^103^Ru/^103m^Rh and ^103^Pd/^103m^Rh *in vivo* generators, while optimizing elution efficiencies and conducting preclinical studies to validate its therapeutic capabilities. Advancing ^105^Rh as a therapeutic radionuclide will require chelators capable of maximizing tumor targeting and minimizing retention, along with scale up of carrier-free routes to enable groundbreaking efficacy and toxicity studies. Streamlining osmium target dissolution and Os/Ir separation—while minimizing ^192^Ir impurities—will be critical in advancing ^191^Os/^191m^Ir generators, while working towards conducting preclinical studies that could translate to clinical utilization. Developing cyclotron-based routes for n.c.a. ^192^Ir with increased specific activity would revolutionize source availability, along with exploring novel complexes that may open new avenues beyond conventional HDR brachytherapy. Both ^97^Ru and ^103^Ru will require continued optimization of production and separation methods to support the design of matched pair theragnostics with further *in vivo* targeting and imaging performance.

In conclusion, we provide a comprehensive review of platinum group metals that have been explored upon over the years, or those that are beginning to make their mark in nuclear medicine applications. These radionuclides offer essential nuclear characteristics that can elevate current areas of necessities, complimenting traditional radionuclides that are utilized in clinical practice. For patients, this could mean more precise imaging options, more effective treatments with fewer side effects, and personalized radiotherapy; therefore, extending the lifespan for someone. As we look ahead, the potential for PGM implementation continues to be promising. With continued efforts from across the world, what was once considered a luxurious dream in nuclear medicine may well become a future breakthrough for diagnostic imaging and cancer therapy with PGMs leading the way.
